# Genome-wide association study identifies genetic risk loci for adiposity in a Taiwanese population

**DOI:** 10.1371/journal.pgen.1009952

**Published:** 2022-01-20

**Authors:** Henry Sung-Ching Wong, Szu-Yi Tsai, Hou-Wei Chu, Min-Rou Lin, Gan-Hong Lin, Yu-Ting Tai, Chen-Yang Shen, Wei-Chiao Chang

**Affiliations:** 1 Department of Clinical Pharmacy, School of Pharmacy, Taipei Medical University, Taipei, Taiwan; 2 Institute of Biomedical Sciences, Academia Sinica, Taipei, Taiwan; 3 Master Program in Clinical Genomics and Proteomics, School of Pharmacy, Taipei Medical University, Taipei, Taiwan; 4 Department of Anesthesiology, Taipei Municipal Wanfang Hospital, Taipei, Taiwan; 5 College of Public Health, China Medical University, Taichung, Taiwan; 6 Department of Pharmacy, Wan Fang Hospital, Taipei Medical University, Taipei, Taiwan; 7 Integrative Research Center for Critical Care, Wan Fang Hospital, Taipei Medical University, Taipei, Taiwan; Stanford University, UNITED STATES

## Abstract

Overweight and obese are risk factors for various diseases. In Taiwan, the combined prevalence of overweight and obesity has increased dramatically. Here, we conducted a genome-wide association study (GWAS) on four adiposity traits, including body-mass index (BMI), body fat percentage (BF%), waist circumference (WC), and waist-hip ratio (WHR), using the data for more than 21,000 subjects in Taiwan Biobank. Associations were evaluated between 6,546,460 single-nucleotide polymorphisms (SNPs) and adiposity traits, yielding 13 genome-wide significant (GWS) adiposity-associated trait-loci pairs. A known gene, *FTO*, as well as two BF%-associated loci (*GNPDA2-GABRG1* [4p12] and *RNU6-2-PIAS1* [15q23]) were identified as pleiotropic effects. Moreover, *RALGAPA1* was found as a specific genetic predisposing factor to high BMI in a Taiwanese population. Compared to other populations, a slightly lower heritability of the four adiposity traits was found in our cohort. Surprisingly, we uncovered the importance of neural pathways that might influence BF%, WC and WHR in the Taiwanese (East Asian) population. Additionally, a moderate genetic correlation between the WHR and BMI (γ_g_ = 0.52; *p* = 2.37×10^−9^) was detected, suggesting different genetic determinants exist for abdominal adiposity and overall adiposity. In conclusion, the obesity-related genetic loci identified here provide new insights into the genetic underpinnings of adiposity in the Taiwanese population.

## Author summary

Overweight and obese individuals are at risk for various medical conditions in Taiwan. Thus, we performed a genome-wide association study (GWAS) on four adiposity traits, including body-mass index (BMI), body fat percentage (BF%), waist circumference (WC), and waist-hip ratio (WHR), using information for more than 21,000 subjects from the Taiwan Biobank. We identified in total 13 genome-wide significant (GWS) adiposity-associated loci, which including well known genetic loci that associated with BMI, such as *FTO*. Additionally, we found *RALGAPA1* as plausible specific susceptibility loci for BMI in Taiwanese. Surprisingly, pathway analysis revealing neuronal implication to BF%, WC and WHR in this population.

## Introduction

Obesity and overweight result from energy imbalances, which cause excessive fat accumulation in the body. Several studies reported that over the past 20 year, the prevalence of obesity has increased [1,2], and if the trends continue, obesity prevalence will reach 18% in men and 21% in women by 2025 [3]. In Taiwan, the prevalence of being overweight and obese (Body-mass index ≥27 kg/m^2^) has grown from 11.8% to 22.0% in the past 25 years (1993~2014) [4]. Body-mass index (BMI) is a common non-invasive indicator of general adiposity, while waist circumference (WC) and the waist-hip ratio (WHR) are measurements of abdominal or visceral fat. Another index, body fat percentage (BF%), reflects fat accumulation in adipose tissues. These measurements associate with various morbidities, such as cardiovascular diseases [5,6], diabetes [5,7,8], and specific types of cancers [9–11].

Family- and twin-based studies revealed that adiposity traits are highly heritable (*e*.*g*., BMI is 40%~70% [12]; WC is 75%, the WHR is 61% [13]; and BF% is 64%~74% [14,15]); thus, genetic factors do play an important role in the development of obesity. Subsequently, large-scale genome-wide association studies (GWASs) and meta-analyses have identified more than 160 loci that are associated with adiposity traits (including the BMI, BF%, WC, WHR, obesity, body weight, and fat distribution) across different populations worldwide [16–22]. Results suggested heterogeneity across adiposity traits, revealing the differences in associated genetic loci or contributions of genetic predispositions. In European studies, BMI-associated loci were enriched in genes that implicated in the central nervous system (CNS) [16]; while WHR-associated loci were enriched in genes that involved in adipocyte metabolism. Furthermore, differences across ancestries were also observed, exemplified by significant enrichment of alcohol metabolism for BMI-associated loci in a Japanese population, but with only a weak implication of a neuronal component [23].

Hundreds of obesity susceptibility loci have been reported in GWAS meta-analyses, however, those studies mainly focused on European ancestries. Here, we conducted a GWAS for adiposity traits (including the BMI, BF%, WC, and WHR) in a Taiwanese population. Based on the findings, we further assessed the relevant cell type, heritability, pathways implicated, and genetic correlations with adiposity. In addition, summary statistics from the UK Biobank (UKBB) GWAS, GIANT consortium, and a Japanese cohort was conducted for replication. Functional annotation was further applied to understand the biological functions of candidate adiposity genes.

## Results

### 13 significant trait-signal pairs were identified for four adiposity traits in a Taiwanese population

In this study, data from 21,978 Taiwanese subjects in the TWB project was analyzed (**Table 1**). The proportions of genders were similar, and the mean age was 48.54±10.98 years. Whole-genome imputation was conducted using a reference panel compiled by Whole genome sequence data of Taiwanese samples and the 1000 Genomes project (1KGP) EAS data, resulting in 14,555,421 SNPs. The consistency rate of the imputation was 99.88%~99.98% (on average for 1,283,000 SNPs). After undergoing stringent quality controls on genotypes and samples (**S1** and **S2 Figs**), we performed a GWAS on four adiposity traits of the BMI (*n* = 21,930), BF% (*n* = 21,304), WC (*n* = 21,949), and WHR (*n* = 21,972) using 6,546,460 SNPs. As shown in S2 Fig, no outliers were detected from the EAS cluster in the PCA. Moreover, O-Q plots and the genomic inflation factor (BMI, λ_GC_ = 1.06; BF%, λ_GC_ = 1.04; WC, λ_GC_ = 1.04; and WHR, λ_GC_ = 1.03) showed no inflation (**S3 Fig**). Therefore, further GC correction of association results was not conducted. The linkage disequilibrium (LD) score intercept of BMI, BF%, WC and WHR were 1.0097±0.0072, 1.0098±0.0067, 1.018±0.0072 and 1.0207±0.0071, respectively.

**Table 1:** Baseline characteristics of individuals included after genotype quality control in the genome-wide association study.

Total (*N*)	21,978				
Males (*N*)	49.85% (10,956)				
Age (range)	48.54±10.98 (30~70) years				
Trait	*N*	Range	Males (mean±SD)	Females (mean±SD)	Total
BMI (kg/m^2^)	21,930	13.56~39.01	25.16±3.38	23.36±3.55	24.26±3.58
BF% (%)	21,304	3.00~56.5	22.89±5.46	31.41±6.29	27.17±7.27
WC (cm)	21,949	50.20~124.00	87.39±9.02	80.14±9.62	83.75±10.0
WHR	21,972	0.61~1.14	0.90±0.06	0.84±0.07	0.87±0.07

BMI, body-mass index; BF%, body fat percentage; WC, waist circumference; WHR, waist-hip ratio; SD, standard deviation.

**Fig 1:**
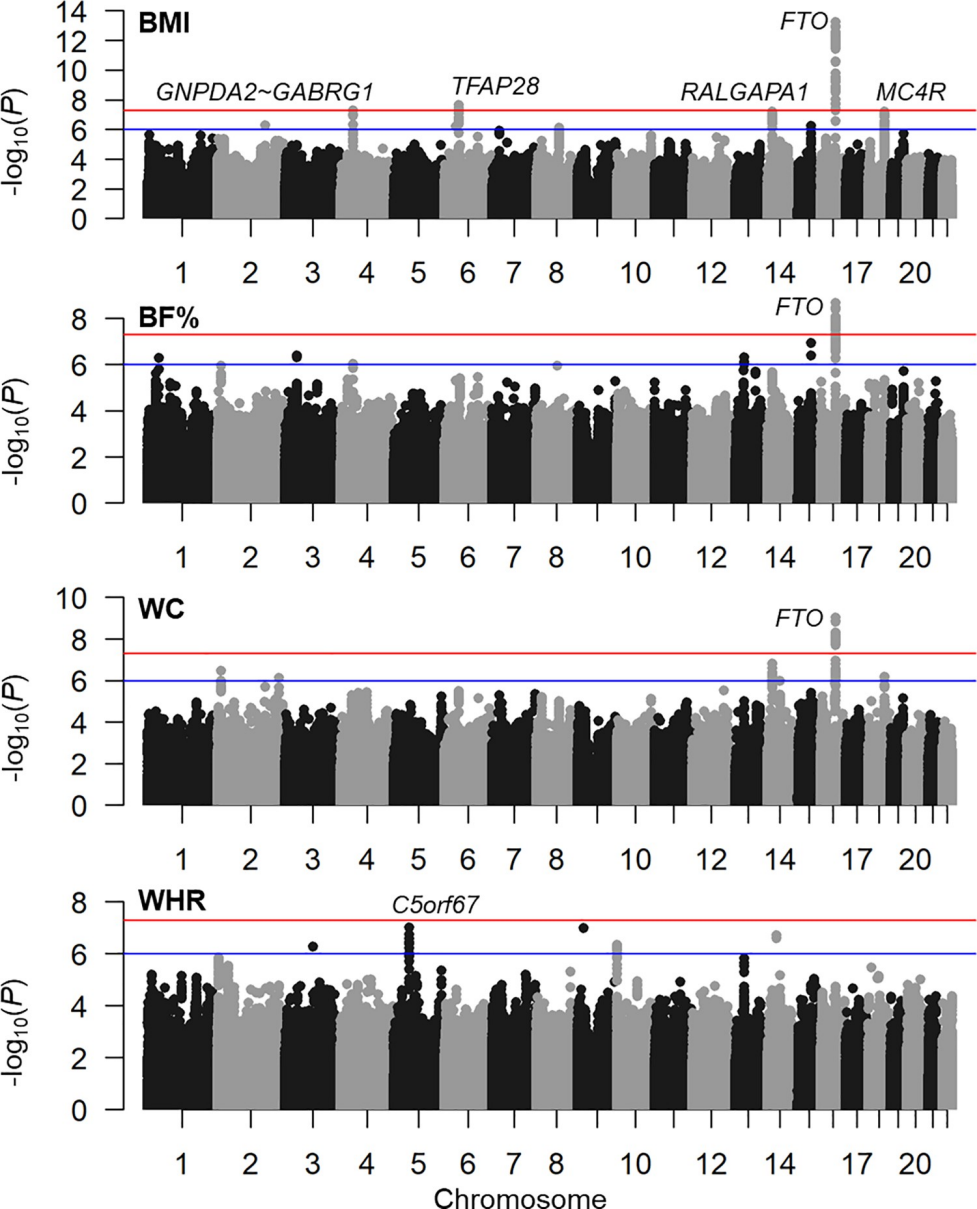
Manhattan plots of adiposity phenotypes. Genetic association results of the body-mass index (BMI), body fat percentage (BF%), waist circumference (WC), and waist-hip ratio (WHR) in a Taiwanese population. Red and blue lines respectively represent genome-wide sequence significance (GWS; *p* = 5×10^−8^) and suggested significance (*p* = 1×10^−6^).

For the four phenotypes (BMI, BF%, WC, and WHR), we respectively detected 130, 53, 54, and 0 SNPs with genome-wide significant (GWS) association signals (*p*<5×10^−8^; **Fig 1** and **S1–S4 Tables**). With the exception of the WHR, genome-wide significance of the *FTO* locus was detected for the remaining three phenotypes. We further conducted a conditional analysis to determine the number of independent signals. By conditioning on the most significant variants of corresponding cytogenetic regions, no additional independent loci for BMI, BF%, or WC was detected (**Table 2** and **S4 Fig**). To annotate the functionality of adiposity trait-associated loci, we further extended the list of suggested significant SNPs with *r*^*2*^≥0.6 and MAF≥0.01. As the result, most of the variants were located in intergenic and intronic regions (85.6%~91.4% across the four phenotypes; **S5 Fig**). We further annotated the epigenetic profiles of these loci and found that over 60% of identified loci (65.8%~81.4%) were assigned a chromatin state across tissues. In each tissue type, nearly 30% of the loci exhibited an open state (ChromHMM state <7; **S6 Fig**), meaning that these loci likely exert biological functions by affecting the transcription.

**Table 2:** Independent genome-wide significant single-nucleotide polymorphisms (SNPs) identified by a conditional analysis.

Trait	SNP	Chr.	Pos.	Alleles	MAF	*β*	SEM	*p* value	Region	PCG(s)
BMI	rs13130484	4	45175691	C/T	0.264	0.059	0.011	5.15×10^−8^	intergenic	*GNPDA2-GABRG1*
	rs141473007	6	50855795	A/G	0.248	-0.062	0.011	2.27×10^−8^	intergenic	*TFAP2B-PKHD1*
	rs8004796	14	36135219	C/T	0.070	-0.101	0.019	5.96×10^−8^	intronic	*RALGAPA1*
	rs1421085	16	53800954	T/C	0.126	0.108	0.014	5.41×10^−14^	intronic	*FTO*
	rs72982988	18	57802714	G/A	0.161	0.072	0.013	6.28×10^−8^	intergenic	*PMAIP1-MC4R*
BF%	rs1421085	16	53800954	T/C	0.126	0.087	0.015	2.16×10^−9^	intronic	*FTO*
WC	rs1421085	16	53800954	T/C	0.126	0.088	0.014	9.36×10^−10^	intronic	*FTO*

Chr., chromosome; Pos., position (based on *Homo sapiens* (human) genome assembly GRCh37 (hg19) from the Genome Reference Consortium); Alleles, were shown in reference/effect; MAF, minor frequency of alternative alleles; *β*, linear regression association coefficient (of alternative alleles); SEM, standard error of the mean; BMI, body-mass index; BF%, body fat percentage; WC, waist circumference. PCG(s), positional candidate gene(s).

The lead variants with *p*<10^−6^ for each phenotype (BMI, *n* = 268; BF%, *n* = 100; WC, *n* = 152; and WHR, *n* = 25) were evaluated by a meta-analysis. Additional GWAS data from the UKBB (denoted UK), GWAS catalog (denoted BBJ, GERA, and Hübel) [23,24], and GIANT consortium (denoted GIANT) was applied [16,25] (**S5 Table**). For a given trait, meta-analyses were separately conducted for each available additional dataset using METAL software. A number of loci reached the genome-wide significance (GWS) threshold for each phenotype, including seven for BMI, two for WC and one for WHR with successful replication rates of 12%~94%. (**S5 Table** and **Table 3**). Accordingly, 13 unique trait-locus pairs were identified for the four adiposity traits in the Taiwanese population.

**Table 3:** The 29 single-nucleotide polymorphisms (SNPs) that were successfully replicated (reached genome-wide sequencing significance (GWS)) by a trans-ethnic meta-analysis and corresponding 13 (significant adiposity-associated) trait-locus pairs.

Trait	Dataset	SNP	Chr.	Pos.	Ref/Alt	MAF	Func. region	Gene	Effect	Std.Err.	*p* value	Dir.	*I* ^*2*^	*P*_*Het*_
BMI	TWB+UK	rs10938397	4	45182527	A/G	0.2638	intergenic	*GNPDA2-GABRG1*	-0.0213	0.0018	3.36×10^−33^	--	91.9	0.000447
BMI	TWB+UK	rs2635727	6	50820940	T/C	0.2285	intergenic	*TFAP2B-PKHD1*	-0.0191	0.002	7.06×10^−21^	--	93	0.000157
BMI	TWB+UK	rs28376697	15	68140315	A/G	0.4419	intergenic	*RNU6-2-PIAS1*	0.0178	0.0021	3.94×10^−17^	++	89.8	0.001757
BMI	TWB+UK	rs11642015	16	53802494	T/C	0.1259	intronic	*FTO*	0.041	0.0018	1.19×10^−114^	++	95.3	3.82×10^−6^
BMI	TWB+UK	rs6567160	18	57829135	T/C	0.181	intergenic	*PMAIP1-MC4R*	-0.0252	0.0021	5.66×10^−34^	--	90	0.001558
BMI	TWB+BBJ	rs13130484	4	45175691	T/C	0.2636	intergenic	*GNPDA2-GABRG1*	0.0315	0.0036	5.64×10^−18^	++	86.2	0.007012
BMI	TWB+BBJ	rs57988840	6	50817748	A/T	0.2275	intergenic	*TFAP2B-PKHD1*	-0.0245	0.0042	4.65×10^−9^	--	91.8	0.000468
BMI	TWB+BBJ	rs78613881	8	81403127	T/C	0.2375	intronic	*ZBTB10*	-0.0275	0.0044	2.57×10^−10^	--	86	0.007505
BMI	TWB+BBJ	rs116994836	14	36127690	T/C	0.07019	ncRNA	*RALGAPA1P1*	-0.0384	0.0065	3.22×10^−9^	--	91.7	0.000506
BMI	TWB+BBJ	rs1421085	16	53800954	T/C	0.1262	intronic	*FTO*	-0.0815	0.0042	2.41×10^−83^	--	73.2	0.05321
BMI	TWB+BBJ	rs6567160	18	57829135	T/C	0.181	intergenic	*PMAIP1-MC4R*	-0.0524	0.0041	3.95×10^−38^	--	0	0.3244
BMI	TWB+GERA	rs10938397	4	45182527	A/G	0.2638	intergenic	*GNPDA2-GABRG1*	-0.0378	0.0025	2.99×10^−53^	--	74.7	0.04681
BMI	TWB+GERA	rs2817419	6	50812906	A/G	0.2318	UTR3	*TFAP2B*	0.029	0.0028	5.70×10^−26^	++	88.6	0.003094
BMI	TWB+GERA	rs16907771	8	81415556	T/G	0.2375	intronic	*ZBTB10*	0.0436	0.0061	8.46×10^−13^	++	37	0.2078
BMI	TWB+GERA	rs8004796	14	36135219	T/C	0.06993	ncRNA	*RALGAPA1P1*	-0.0807	0.0135	2.41×10^−9^	--	59.8	0.1149
BMI	TWB+GERA	rs4776990	15	68137364	T/C	0.4439	intergenic	*RNU6-2-PIAS1*	-0.0283	0.003	1.23×10^−21^	--	78.7	0.03042
BMI	TWB+GERA	rs1558902	16	53803574	A/T	0.1258	intronic	*FTO*	0.0798	0.0025	6.48×10^−222^	++	72	0.05861
BMI	TWB+GERA	rs6567160	18	57829135	T/C	0.181	intergenic	*PMAIP1-MC4R*	-0.0528	0.0029	3.40×10^−75^	--	0	0.3574
BF%	TWB+UK	rs10938397	4	45182527	A/G	0.2638	intergenic	*GNPDA2-GABRG1*	-0.0212	0.0018	1.02×10^−32^	--	89.1	0.002493
BF%	TWB+UK	rs28376697	15	68140315	A/G	0.4419	intergenic	*RNU6-2-PIAS1*	0.0179	0.0021	3.14×10^−17^	++	90.9	0.000907
BF%	TWB+UK	rs11642015	16	53802494	T/C	0.1259	intronic	*FTO*	0.0406	0.0018	1.10×10^−112^	++	89.7	0.001811
BF%	TWB+Hübel	rs10938397	4	45182527	A/G	0.2638	intergenic	*GNPDA2-GABRG1*	-0.085	0.0098	3.35×10^−18^	--	97.4	6.65×10^−10^
BF%	TWB+Hübel	rs28376697	15	68140315	A/G	0.4419	intergenic	*RNU6-2-PIAS1*	0.0606	0.0091	3.32×10^−11^	++	90.1	0.001512
BF%	TWB+Hübel	rs1421085	16	53800954	T/C	0.1262	intronic	*FTO*	-0.1604	0.0121	2.02×10^−40^	--	98.8	2.46×10^−19^
WC	TWB+UK	rs11642015	16	53802494	T/C	0.1259	intronic	*FTO*	0.0573	0.0021	5.26×10^−167^	++	77.3	0.03597
WC	TWB+UK	rs9954728	18	57801557	C/G	0.1802	intergenic	*PMAIP1-MC4R*	-0.031	0.0022	3.88×10^−46^	--	84.6	0.01091
WC	TWB+GIANT	rs9939973	16	53800568	A/G	0.1702	intronic	*FTO*	0.0725	0.0033	6.16×10^−108^	++	0	0.5501
WC	TWB+GIANT	rs1942880	18	57793209	T/C	0.1795	intergenic	*PMAIP1-MC4R*	0.0362	0.0035	1.45×10^−25^	++	79.2	0.02828
WHR	TWB+GIANT	rs459193	5	55806751	A/G	0.47	downstream	*C5orf67*	0.0288	0.0035	3.11×10^−16^	++	75.2	0.04455

Chr., chromosome; Pos., position; Ref, reference allele; Alt, effect allele; MAF, minor frequency of alternative alleles; Effect, meta-analysis-estimated effect size; Std.Err., standard error for effect size estimate; Dir., the effect direction of each study; *I*^*2*^, heterozygosity (%); *P*_*Het*_, *p* value of the heterozygosity statistic (Chi-squared distribution with a degree of freedom of 1); BMI, body-mass index; BF%, body fat percentage; WC, waist circumference. Gray, leading SNPs (with the highest *p* values) of unique associated loci.

### Four phenotypes shared similar adiposity-associated loci

For adiposity trait-associated loci, four (of 13; 30.77%) variants (represented by rs6567160 [BMI], rs10938397 [BMI and BF%], rs11642015 [BF% and WC], and rs1558902 [BMI]) exhibited pleiotropy, with between three and 19 reported associations per variant (**S6 Table**). Notably, all four SNPs have been reported to associate with BMI and obesity according to GWAS catalog database (extracted on November 11, 2020). In addition, most of these loci (three of four; 75%) were identified for early-onset extreme obesity, age onset of menarche, and high-density lipoprotein (HDL) cholesterol. In this study, we also detected a locus associated with height (represented by rs6567160) [26] and two loci associated with type 2 diabetes (T2D; represented by rs11642015 and rs1558902) [23] that were consistent with results in a Japanese population. Surprisingly, we identified two unreported loci (the *GNPDA2-GABRG1* locus represented by rs10938397 [chr4:45182527; cytoband 4p12; hg19] and the *RNU6-2-PIAS1* locus represented by rs28376697 [chr15:68140315; cytoband 15q23; hg19]) for BF%. Since no associated signal for BF% had previously been identified on chromosome 4 or 15, we did not apply LD checking to the two novel loci or confirm the independence of these loci to those previously reported BF%-associated loci. The rs10938397-A (risk) allele exhibited the higher frequencies in East Asians and Taiwanese (risk allele frequencies [RAFs] = 0.697~0.731) compared to European populations (RAFs = 0.561~0.58; based on the 1KGP and the genome Aggregation Database [gnomAD]; **S7 Table**). Similarly, the rs28376697-G (risk) allele also showed the higher frequencies in East Asians and Taiwanese population (RAFs = 0.473~0.544) compared to European populations (RAFs = 0.188~0.226). Both of these BF%-associated variants were intergenic. We next annotated variants in LD (*r*^*2*^>0.8; from the TWB) with two variants, resulting in 12 additional variants for rs10938397 and four additional variants for rs28376697 (**S8 Table**). These 18 [*i*.*e*., (12+1)+(4+1)] SNPs were all in intergenic regions.

### Functional annotations for adiposity-associated SNPs

Since none of these SNPs was located in an exonic region, functional annotations (FAs) of the 18 SNPs were thus inspected based on the HaploReg (v4.1) [27] and RegulomeDB [28] databases (**S9 Table**). Using 11 different FA criteria, we observed that one (9.09%) to seven (63.64%) of the 11 FAs were available for the 18 SNPs, suggesting biological relevance (through transcriptional or epigenetic regulation) of these SNPs to the susceptibility of BF% trait. Finally, we justified that these loci were an endorsement of genetic pleiotropy of adiposity traits as follows: (1) These two loci were also associated with the BMI (based on previous GWASs and this study); (2) Both these SNPs were associated with a wide range of adiposity-related traits based on the UK Biobank (*e*.*g*. trunk fat mass, impedance, and critically BF% by limb).

### RALGAPA1 as plausible specific susceptibility loci for BMI in the Taiwanese population

**Fig 2:**
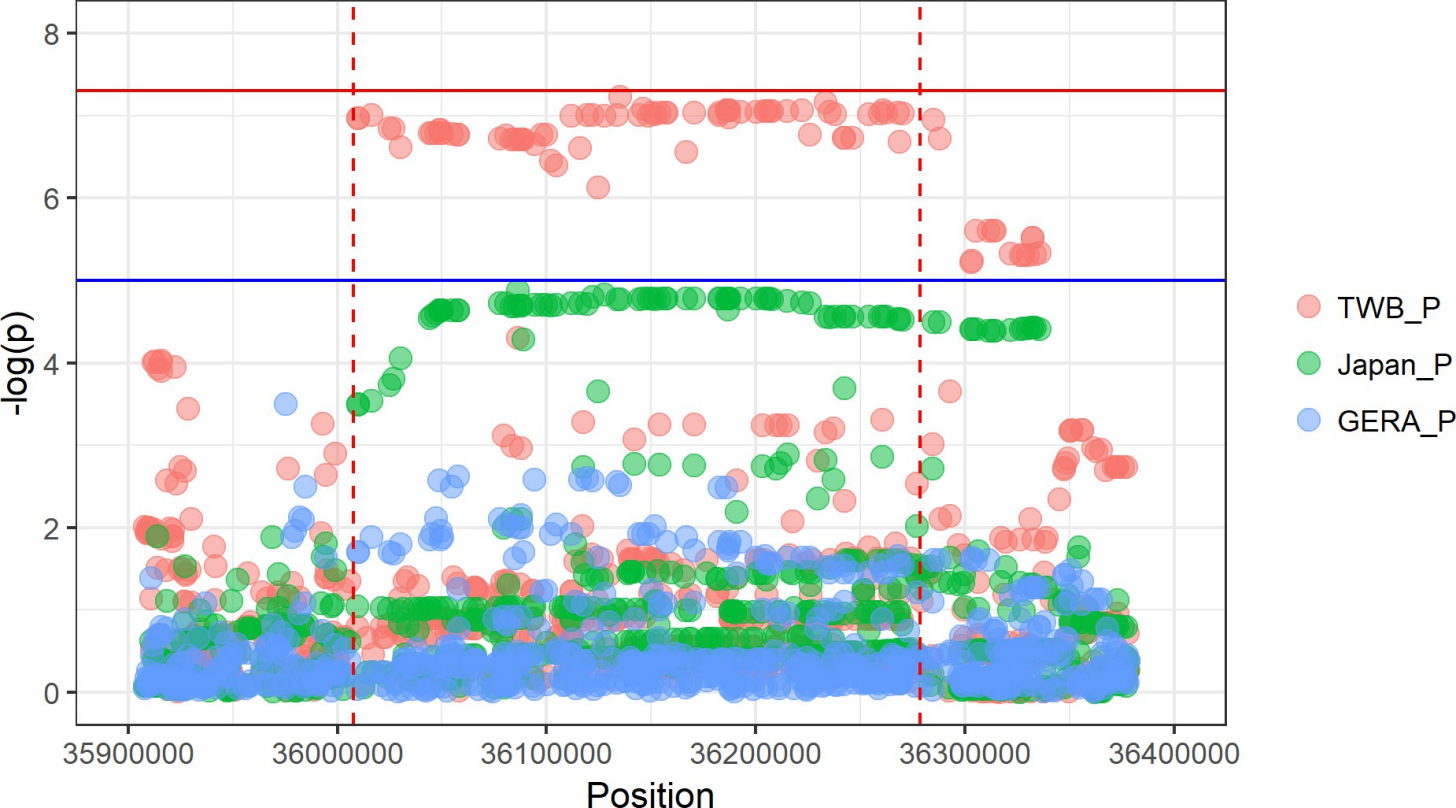
A novel gene, *RALGAPA1*, identified by comparison of Taiwan Biobank (TWB), GERA and Japan Biobank (BBJ) genome-wide association studies (GWASs). The *X*-axis represents the position of loci (hg19). The *Y*-axis represents -log_10_(*p*). Red dots are *p* values of variants from the TWB. Green dots are *p* values from the BBJ. Blue dots are *p* values from GERA cohort (GERA). The blue horizontal line represents *p* = 1×10^−5^. The red horizontal line represents *p* = 5×10^−8^. The red dashed line represents the location of the gene.

Based on our results, a GWS signal for *RALGAPA1* (within cytoband 14q13.2) was identified to correlate with BMI. However, only one significant BMI signal (rs10140922) within ±1-Mb flanking region of the top signal (rs8004796) in *RALGAPA1* has previously been reported [25]. In addition, the SNPs were not in LD (*r*^*2*^<0.1 in African, American, European, East Asian and South Asian based on 1000 Genomes, Phase 3 v5). We further narrowed down this region, however, no significant signal was found in either the GERA BMI [17], BBJ BMI [23], or GWAS catalog (BMI trait) within a ±100-kb region of *RALGAPA1* (**Fig 2**). FAs revealed biological implications of *RALGAPA1* for BMI, BF%, and WC (but not WHR), which included (1) a Combined Annotation-Dependent Depletion (CADD) score of >12.37; (2) 15-core chromatin state mapping; and (3) 3D chromatin interaction mapping (**S7 Fig**). Moreover, to assess the frequency of variants near *RALGAPA1* across different populations, we retrieved SNPs within a ±100-kb region to the gene from 1000G Database. As a result, the MAF of SNPs near the *RALGAPA1* locus was higher in a Taiwanese population than that in Caucasians (SNP MAF: ~0.07 *vs*. ~0.001; **S10 Table**). Taken together, we suspected that *RALGAPA1* is a specific adiposity-susceptibility gene to the Taiwanese population.

### Cell types associated with adiposity traits in a Taiwanese population

**Fig 3:**
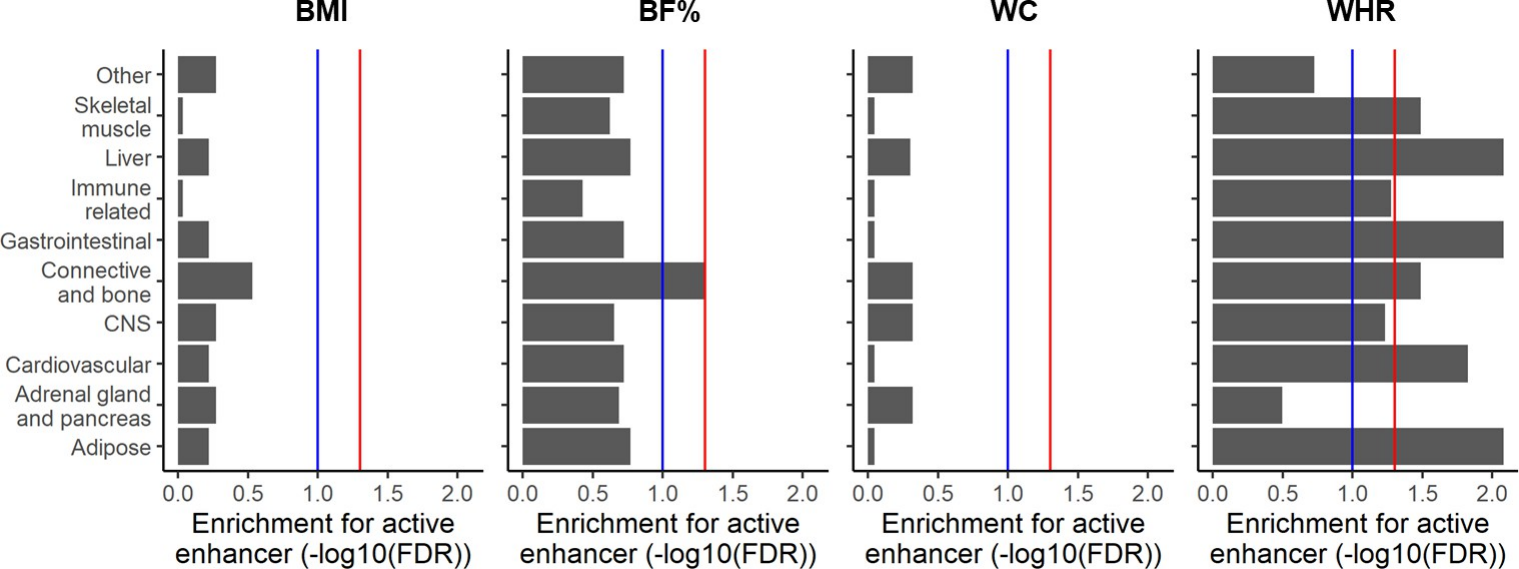
Active enhancer enrichment of obesity-related variants in 10 cell type categories. *p* values were obtained from 2000 permutations. The false detection rate (FDR) was obtained using the Benjamin-Hochberg method. The red line denotes an FDR of <0.05, and blue line denotes an FDR of <0.1.

To identify critical cell types that are genetically associated with the four adiposity traits in a Taiwanese population, we conducted cell type enrichment tests on Taiwanese GWAS results using active enhancer profiles of 10 cell type categories from the Roadmap project. To avoid confounding due to the variant LD patterns and allelic frequency differences, we first extracted a subset of variants that reached to the suggested significance by LD clumping. Then, we performed a permutation by controlling the MAF of background variants. For BF%, significant enrichment of variants in active enhancers was observed in the connective bone category (FDR = 0.05; **Fig 3**). Further investigation revealed nominally significant enrichment of active enhancers of all three cell types from the connective bone category (including connective cells, fibroblasts, and osteoblasts; all *p*<0.05; **S8 Fig**). For the WHR, we detected enrichment in six (of 10) categories (including adipose, cardiovascular, connective and bone, gastrointestinal, liver, and skeletal muscle; with FDRs of <0.05). In addition, no enrichment of BMI- or WC-related variants of active enhancers was detected in any of the 10 cell categories.

A previous Japanese study identified the enrichment of the BMI in B cells’ active enhancers (a subgroup of the “immune-related” category) [23], however, we are not able to replicate that observation (all nominal *p*>0.05; **S9 Fig**). Here, we detected nominal significance enrichment of WHR-related variants of active enhancers of the spleen and thymus. Moreover, studies suggested a role of the CNS in the BMI [29,30]. However, we found no evidence regarding neuronal implications in the BMI in our Taiwanese population (**Fig 3**). Subgroup active enhancer enrichment analyses were further conducted by categorizing the CNS category into seven subgroups. As a result, we found significant enrichment (with an FDR of <0.05) in active enhancers of the anterior caudate, cingulate gyrus, and inferior temporal lobe for BF% (**S10 Fig**); and suggested significant (with an FDR of <0.1) of the middle hippocampus (for BMI and BF%), anterior caudate (for BMI), anterior caudate (for BMI), cingulate gyrus (for BMI), inferior temporal lobe (for BMI), and angular gyrus (for BF%). In contrast, enrichment was not detected of any of the seven neuronal subgroups for WC or the WHR.

### Variance explained for four adiposity traits by GWAS variants in a Taiwanese population

The polygenic effects of the four phenotypes were estimated by all GWAS variants in our Taiwanese population-based cohort. Autosomal variants accounted for a proportion of 23.94%±1.6% (standard error of the mean (SEM)) of the phenotypic variance of BMI. This proportion was slightly lower than those of Europeans (27.4%±2.5%) [31] and Japanese (29.8%±3.4%) [23]. However, the proportion of variance explained by the BMI was comparable to the UKBB GWAS (24.6%). For BF%, WC, and the WHR, estimated phenotypic variances explained by autosomal variants were 18.35%±1.7% (*vs*. the UKBB GWAS of 22.0%), 18.47%±1.6% (*vs*. the UKBB GWAS of 20.4%), and 13.41%±1.6% (*vs*. Scotland of 24.0%±1.0% [32]), respectively. Overall, we observed a slight decrease in the proportions of variance explained by adiposity traits in a Taiwanese population.

### Pathway analysis for the loci associated with adiposity traits

Using lead variants for each phenotype from Taiwanese subjects, we conducted pathway analyses to identify canonical pathways (CPs) or gene ontology (GO) biological pathways (BPs) for adiposity traits. Using an FDR threshold of 0.05, we found that variants associated with the BMI were all implicated in neuron-related pathways (**Table 4**). These results were similar to the previous findings in Europeans [16], *i*.*e*., the BMI contains a neuronal component. Surprisingly, we detected neuronal pathway associations for variants associated with BF%, WC, and the WHR, suggesting neuronal implications in adiposity in the Taiwanese population. For the WHR, additional pathways were identified, which included an estrogen receptor (non-genomic) pathway, potassium channels, platelet homeostasis, glycoaminoglycan degradation, and a developmental pathway, which implied more-complicated biological regulation of the WHR compared to the BMI, BF%, and WC.

**Table 4:** Significant pathways (with a false discovery rate (FDR) of <0.05) that were associated with adiposity traits.

Trait	Canonical pathways	Gene ontology biological processes
BMI	*N*.*s*.	1. Nervous system development
		2. Neuron differentiation
		3. Generation of neurons
		4. Central nervous system development
		5. Neurogenesis
BF%	*N*.*s*.	1. Generation of neurons
		2. Neurogenesis
		3. Nervous system development
		4. Central nervous system development
		5. Neuron differentiation
WC	[Kegg] Axon guidance	1. Generation of neurons
		2. Neurogenesis
		3. Neuron differentiation
		4. Nervous system development
		5. Neuron development
		6. Axonogenesis
		7. Neurite development
		8. Cellular morphogenesis during differentiation
WHR	1. [Reactome] Neuronal system	1. Anatomical structure development
	2. [Reactome] Axon guidance	
	3. [Reactome] Developmental biology	
	4. [PID] ER nongenomic pathway	
	5. [Reactome] transmission across chemical synapses	
	6. [Reactome] potassium channels	
	7. [Reactome] platelet homeostasis	
	8. [Kegg] glycosaminoglycan degradation	

*N*.*s*., no significance; [Kegg]: Kyoto Encyclopedia of Genes and Genomes; [Reactome]: Reactome Pathway Database; [PID]: The Pathway Interaction Database. The pathways were ordered by significance (from the most significant to the less significant pathways).

### Genetic correlation analysis among adiposity traits

Due to similar results obtained for adiposity phenotypes, we further identified genetic correlations among the BMI, BF%, WC, and WHR. Our results showed that the BMI exhibited high genetic correlations with BF% and WC, indicating that the pleiotropic effects of genes control three phenotypes. For example, WHR showed a modest correlation (γ_g_ = 0.5~0.7) with BMI, BF% and WC, meaning that genes affecting central obesity are not as the same as those affecting general obesity (**S11 Fig**). Our findings were consistent with previous studies in Asian population (genetic correlation of 0.53 between BMI and WHR) [33] and the National Heart, Lung, and Blood Institute Twin Study (genetic correlation of 0.52 between BMI and WHR) [34].

## Discussion

Here, we revealed 13 independent adiposity phenotypes-related trait-loci pairs (seven for BMI, three for BF%, two for WC, and one for WHR) by integrating medical data and genome-wide SNP profiles from TWB. These associations were successfully replicated in a meta-analysis of Japanese and European populations. Based on our association analysis and LD-based clumping results, *FTO* loci strongly associated with BMI, BF% and WC. *FTO* is widely known to be associated with obesity from previous reports on different ethnic groups [17], and it has been strongly associated with several adiposity phenotypes, such as extreme obesity [35], early-onset obesity [35], the BMI [36], BF% [24], WC [25], and the WHR [25]. In this study, rs1421085 (located in the *FTO* locus) was associated with the BMI, BF% and WC (**Tables 2** and **3**). Actually, this particular SNP was reported to increase expression of *IRX3* and *IRX5* through 3D chromatin effects during preadipocyte differentiation, thereby enhancing the formation of energy-storing white adipocytes, lipid storage, and obesity development [37]. Interestingly, when we compared the MAF of rs1421085 in our cohort with those from the UKBB and BBJ cohorts, we found a lower MAF of rs1421085 (of 0.126) in the Taiwanese population than that in European (MAF of 0.402) and Japanese (MAF of 0.201) populations. Notably, we found no significant associations between the *FTO* gene and the WHR trait in our study population. Since the top BMI variant (exemplified by rs11642015) in the *FTO* gene showed a *p*-value of 1.842×10^−5^ in WHR (TWB data), we denoted that this finding may be due to lack of statistical power. Other loci that were significantly associated with the BMI in this study including rs13130484 on 4p12 [23,38], rs141473007 on 6p12.3 [17], and rs72982988 on 18q21.32 [17,23], which were also significantly associated with obesity in European and East Asian populations. By comparing the SNPs identified here with cataloged GWAS reports, we evaluate the pleiotropic effects of genes on adiposity phenotypes. Four loci were associated with multiple adiposity phenotypes, such as BMI, early-onset extreme obesity, fat body mass, and WC, among others (**S3 Table**). These results suggested that pleiotropic effects of some genes lead to several adiposity phenotypes. For example, rs109383397 may simultaneously impact BMI and BF%. Moreover, loci in *FTO* (*e*.*g*., rs11642015 and rs1558902) exhibited pleiotropic effects on T2DM and metabolic syndrome, indicating that these loci may have effects on obesity-related comorbidities. In line with these findings, a genetic correlation analysis revealed strong similarities among BMI, BF% and WC phenotypes, suggesting that the genes-mediated phenotypes are highly correlated. Unexpectedly, WC, an indicator of abdominal obesity, showed higher genetic correlations with BMI (γ_g_ = 0.88) and BF% (γ_g_ = 0.9); meanwhile, another indicator of abdominal obesity, WHR, showed a moderate genetic correlation with BMI (γ_g_ = 0.52) but a high genetic correlation with WC (γ_g_ = 0.7), consistent with the findings of a previous study [39]. Notably, we observed higher correlations among BMI, BF% and WC, and relatively lower genetic correlations with WHR. This observation was further supported by the pathway analysis, which revealed that BMI, BF% and WC were only associated with neural processes; however, in WHR was associated with pathways including estrogen receptor (non-genomic) signaling, potassium channels, platelet homeostasis, glycoaminoglycan degradation, and a developmental pathway.

In this study, we found that the *RALGAPA1* locus are specifically associated with BMI in the Taiwanese cohort. SNPs in *RALGAPA1* were successfully replicated in a meta-analysis on East Asian (BBJ cohort) and multi-ethnic populations (GERA cohort); 92 SNPs were with the suggested significance level in the ±100-kb region around *RALGAPA1*. We also examined the *cis*-expression quantitative trait locus (*cis*-eQTL) data of the most significant SNP, rs8004796, and found the *cis*-eQTL in adipose-visceral tissues (*p* = 0.0032; FDR = 0.000053). Aside from the *cis*-eQTL evidence, we also found H3K4me3 histone modifications and a transcription factor-binding site (RBM22) at rs8004796 by using UCSC genome browser. However, there was no evidence of transcriptional activities in a cell line (**S12 Fig**). We further examined another SNP, rs76115093, which exhibited high LD with rs8004796 (*r*^*2*^ = 1). Indeed, several transcription factor-binding sites were predicted around rs76115093. The functional experiments showed the activity of a CCCTC-binding factor (CTCF), therefore, we considered rs76115093 to be a potentially functional variant that might alter *RALGAPA1* expression via a 3D interaction with the promoter region (**S13 Fig**) [40]. These results imply that fluctuations in gene expression levels may be linked to the rs8004796 polymorphism. Furthermore, MAFs of SNPs in *RALGAPA1* were low (**S7 Table**). We considered the association between this gene and BMI to be specific to the Taiwanese population. Previous studies reported that overexpression of the RALGAPA1 mutant protein (Thr735Ala) decreases RalA protein activation, and it attenuates RalA-dependent glucose transporter 4 (GLUT4) and cluster of differentiation 36 (CD36) translocation in response to insulin stimulation in skeletal muscle cells [41]. Thus, further testing will be needed to determine whether the RALGAPA1 is associated with obesity in an independent Taiwanese cohort.

A heritability analysis revealed a slight decrease in the proportions of variance explained by all four adiposity traits in the Taiwanese population compared to that in Europeans and Japanese populations. These results confirmed the existence of genetic predispositions to adiposity traits and highlighted the importance of environmental determinants. Based on these findings, it is most reasonable to consider environmental interventions (healthy lifestyle promotions, weight-loss plans, promotion of regular exercise, *etc*.) for managing or controlling adiposity in different populations. In accordance with this point of view, Lin et al., reported that regular exercise (especially regular jogging) can diminish BMI, BF%, WC and HC [42]. Importantly, promotion of regular physical exercise is most salient to Taiwanese subjects with a greater genetic predisposition to adiposity.

A previous study on BMI-associated genes in a European population nicely showed enrichments in central nervous system-related processes, including the involvement of neurotrophin signaling pathway, synaptic function, and glutamate signaling [16]. On the other hand, ethanol oxidation and glycolysis gluconeogenesis pathways were identified in an East Asian population [23]. For WC and WHR phenotypes (both BMI-adjusted), genes identified in Europeans were found to be significantly enriched in adipose-related tissues, adiponectin signaling and insulin sensitivity [25], whereas the genes in East Asians were enriched in corticotropin-releasing hormone signaling and gonadotropin-releasing hormone (GnRH) signaling pathways [18]. These results suggest a need to assess multiple populations in order to better understand the pathways underlying adiposity. In the current study, we found that all loci associated with the four adiposity traits were significantly enriched in neuron-related pathways (**Table 4**), however, these pathways were not reported to be associated with BF%, WC or WHR in previous studies. For BF% in particular, this finding was further supported by results of an active enhancer enrichment analysis (**S10 Fig**), which showed significant enrichment in active enhancers of BF%-associated loci for the anterior caudate, cingulate gyrus, and inferior temporal lobe. We also found enrichment of WHR-associated genes in a developmental pathway, which is consistent with previous findings [25]. Thus, our results revealed adiposity-associated pathways that have particularly pronounced effects on adiposity in a Taiwanese population.

There are some limitations in this study. First, we did not observe a GWAS signal for WHR in our cohort (before the meta-analysis), probably because the sample size used for the analysis was not enough to obtain sufficient power. Second, we accounted for age, age^2^, sex, and the top 10 PCs of ancestry as covariates, whereas many other environmental factors may be associated with adiposity phenotypes, such as current medications, physical activity levels, smoking behavior, and alcohol usage. Gene-environment effects should be considered and estimated in future studies.

In this study, we performed a GWAS in a Taiwanese population and identified 13 significant adiposity-associated trait-locus pairs. We revealed two BF% loci (*GNPDA2-GABRG1* [4p12] and *RNU6-2-PIAS1* [15q23]) that were pleiotropic across adiposity-related traits and uncovered a BMI-associated locus in *RALGAPA1*, which is specific to the Taiwanese population. A heritability analysis revealed a slightly lower than expected variance of adiposity traits that could be explained by genetic predispositions, suggesting the importance of behavioral interventions to improve adiposity. Finally, our results revealed the critical role of neural effects on BF%, WC and WHR.

## Materials and methods

### Ethics statement

Ethical approval of the study was granted by the Institutional Review Board (IRB no. N201802059) of Taipei Medical University and TWB, Academia Sinica (TWBR10505-05, TWBR10602-02, TWBR10703-02, and AS-IRB01-16018) before conducting the study.

### Study population

This study included 23,996 subjects with complete information of age, sex, BMI, and whole genotype data from the Taiwan Biobank (TWB). To date, 114,533 participants have been recruited from the community, containing a total of 27,700 genome-wide array genotyping data among them. The main aim of the TWB is to provide researchers with health information of Taiwanese people so that they can improve health-related problems concerning common chronic diseases. Previously, Chen *et al*. [43] and Lo *et al*. [44] demonstrated that ~80% of Han Taiwanese are genetically closer to the Southern Han ancestry in China. However, since the study subjects were all collected from the Taiwanese cohort, we use the term “Taiwanese” for precision sake.

Local community participants that are included were aged 30~70 years, were physically active, and were self-reported to be of Taiwanese Han-Chinese ancestry; specimens, blood, urine, detailed questionnaires, and data of a physical examination were collected. Written informed consent was obtained from all the individuals while participating in the TWB project. Participants were all genotyped by a custom Axiom Genome-Wide Array Plate, that contained 646,973 single-nucleotide polymorphism (SNP) sites, called the TWB chip 1, based on technology developed by Affymetrix. Individuals with cancer or a cancer history, who were non-residents of Taiwan, or who withdrew were excluded. In total, 21,978 subjects were included for a subsequent analysis after quality control.

### Data quality control steps and imputation

For sample quality control of the GWAS, samples with ambiguous sex data (by sex check) were excluded; individuals with a call rate of <0.95, related individuals (PIHAT >0.1875), and those with a heterozygosity rate of greater than the mean±5 standard deviations (SDs); calculated by PLINK v1.9 [45]) were further excluded. Moreover, all samples passed the ancestry check. For genotypic quality control, SNPs with a call rate of >0.05, Hardy-Weinberg equilibrium (HWE) *p*-value of <1×10^−5^ (calculated using only control subset), and a minor allele frequency (MAF) <0.05 were further discarded.

Genotype imputation was performed using SHAPEIT (v2.r790) [46] and IMPUTE2 (v2.3.1) [47], with a reference panel compiled by a Taiwanese population (TWB) and 1000G East Asian (EAS) population whole-genome sequencing (WGS) data. After imputation, multi-allelic sites and SNPs with a post-imputation call rate of >0.05, a MAF of <0.01, or *R*^*2*^<0.3 were excluded. The final set of 6,546,460 variants were prepared for the following association analysis.

To assess the population stratification of our Taiwanese population, we merged the data using ~60,000 SNPs (with low linkage disequilibrium (LD) (*r*^*2*^<0.2) and MAFs of <0.05) to 1KGP data with 26 populations. Principal component analysis (PCA) was then conducted using GCTA [48]. As no individuals were outside of the East Asian cluster, we included all individuals in subsequent analyses.

### Phenotype preparation

We collected data (height, weight, WC, hip circumference (HC), and BF%) from physical examination data in the TWB. The BMI was calculated as the weight (kg) divided by the height squared (m^2^); BF% was estimated by a bioelectrical impedance analysis (BIA); the WHR was a comparison of WC (cm) and HC (cm). Outliers were defined as a phenotypic value beyond the mean±4 SDs. There were 21,930 individuals with BMI data; 21,304 individuals with BF% data; 21,949 individuals with WC data; and 21,972 individuals with WHR data. All phenotypes (BMI, BF%, WC, and WHR) were further standardized using a rank-based inverse transformation method because of right-skewed distributions. To do this, we first obtained residuals for log-transformed phenotypes using a linear regression analysis which was adjusted for age, age^2^, sex, and top 10 PCs of ancestry. Then, we transformed the residuals via a rank-based inverse transformation.

### Association analysis

An SNP-based association analysis was performed using PLINK (v1.9) [45] based on linear regression. Owing to the previous adjustment of age, age^2^, sex, and top 10 PCs of ancestry during phenotype preparation, we did not perform any further adjustment in the association analysis. Manhattan plots and quantile-quantile (Q-Q) plots were generated with the *qqman* package.

### LD score regression analysis

The LD score intercepts of the four adiposity traits in Taiwanese population were calculated using the LDSC package [49].

### Conditional analysis and meta-analysis

A conditional analysis was conducted using GCTA [50] to determine independent signals near the most significantly associated loci (±1-Mb region). LD information was calculated using imputed data of all individuals, and SNPs with collinearity of >0.9 were excluded. We applied LocusZoom to generate regional association plots [51]. A meta-analysis was performed using the inverse-variance fixed-effect method implemented in METAL software [52] with no further genomic control (GC) correction. Heterogeneity between studies was calculated with Cochran’s *Q* test. Successful replication *p* was designated at 5×10^−8^, with directions consistent between the discovery and replication datasets.

Summary statistics were downloaded from the GWAS catalog [53], GIANT consortium, and UK Biobank (UKBB) as follows: (i) ***The GIANT consortium***. For WC and WHR, we retrieved the data from Shungin, D. *et al*. [25] that contained 224,459 individuals. In total, 1109 and 544 signals for WC (top variant: rs1558902; *FTO* gene) and WHR (top variant: rs1121980; *FTO* gene) were identified in the study, respectively. (ii) ***GWAS catalog***. Rank-based inverse transformed BMI data from a Japanese cohort conducted by Akiyama *et al*. [23] were used in our study. This cohort contained 173,140 Japanese individuals (Biobank Japan; BBJ) and 5,961,601 variants. In total, 5903 variants reached the genome-wide significance threshold, with 112 novel loci against the previous studies being identified. The strongest signal was rs11642015 on the *FTO* gene. A multi-ethnic BMI (rank-based inverse transformed) cohort [17] contained 334,487 individuals (non-Hispanic whites, Latinos, East Asians, African Americans and South Asians) was also included in this study. With 10,694 genome-wide significant signals identified, 30 novel loci were further identified. The most significant locus was rs1421085 on the *FTO* gene. Additionally, BF% data was obtained from a study conducted by Hübel C *et al. [24]*, which contains 155,961 Europeans. In this study, 5,056 variants reached the genome-wide significant threshold, with the strongest signal be rs1421085 on the *FTO* gene. (iii) ***The UK Biobank***. Summary statistics of four adiposity-related traits (rank-based inverse transformed; 361,194 samples for each phenotype) were acquired from Neale’s lab website (http://www.nealelab.is/uk-biobank). The number of significant loci in BMI, BF%, WC and WHR were 39,879, 39,879, 31,847 and 35,029, respectively. For BMI, the most significant signal was rs62048402 (*FTO* gene). For BF% and WC, the top signal was rs11642015 (*FTO* gene). For WHR, the top signal was rs577721086 (*FTO* gene). Due to the incompleteness of genotype information in WHR, we thus directly compared the regression *β* values between WHR summary statistics and our suggestive significant loci using Spearman’s correlation test.

### Functional annotations for variants

Independent significant SNPs with each other at *r*^*2*^<0.1and SNPs with high LD of these SNPs (*r*^*2*^≥0.6) were mapped to genes in FUMA v1.3.4 [54] using the following strategies. (1) *Positional mapping*. FUMA performed positional mapping based on SNP annotation from ANNOVAR [55] using a default physical distance (of ±10 kb). Then the lists of SNPs were further mapped with the Combined Annotation-Dependent Depletion (CADD) score. (2) *Chromatin state mapping*. To investigate the potential regulatory functions of our loci, we applied chromatin state mapping based on the 15-core ChromHMM model. The chromatin state with 15 categorical states represented the genome accessibility predicted by ChromHMM based on epigenomic data of five chromatin marks (H3K4me3, H3K4me1, H3K36me3, H3K27me3, and H3K9me3) for 127 cell types. (3) *3D chromatin interaction mapping*. FUMA maps SNPs to genes based on Hi-C data (GSE87112) of 21 tissues/cell types [56]. The default threshold of the false discovery rate (FDR; *p*<10^−6^) was set to define significant interactions.

### Cell type specificity analysis

Active enhancer profiles of different cell types in the ChromHMM 18-state model (state 9) were acquired from the Roadmap project. We first grouped cell types into 10 cell type categories for analysis. Clumped SNP lists of the four adiposity traits were subjected to active enhancer enrichment tests. The test was conducted by comparing the number of overlapping clumped variants in active enhancers to the number of overlapping randomly selected variants in active enhancers. To avoid bias from LD, the background SNP list was pruned based on LD information of Taiwanese population genomic profiles. To prevent confounding from allele frequency differences, a background gene list was sampled based on allele frequency categories. The allele frequency categories were as follows: (1) category 1: MAF≤5%; (2) category 2: 5%<MAF≤15%; (3) category 3: 15%<MAF≤30%; and (4) category 4: MAF>30%. For each test, we repeated the sampling procedure 2000 times (permutations) to calculate *p* values. The FDR was finally estimated using the Benjamini-Hochberg (B-H) method.

### Heritability analysis

To determine the proportion of variance explained (heritability) with autosomal variants, we first generated a genetic relationship matrix (GRM) using genome-wide complex trait analysis (GCTA)-genomic restricted maximum likelihood (GREML) method. The log-transformed phenotype value was transformed to a *Z* score and adjusted for age, age^2^, and sex. An estimate of the variance of adiposity phenotypes was then calculated using the “reml” function implemented in GCTA with parameter “*—grm-cutoff 0*.*05*”.

### Pathway analysis

To determine which mechanisms are involved in regulating adiposity on our gene lists, we conducted a pathway analysis using the *snpGeneSets* package. The annotation file (Homo_sapiens.GRCh37.87.gtf) for SNP-to-gene mapping was acquired from the website ftp://ftp.ensembl.org/pub/grch37/current/gtf/homo_sapiens/. In this study, the SNP-to-gene mapping step was conducted by setting the flanking region to 2000 bp.

### Genetic correlations and pleiotropic analysis

To estimate genetic correlations (γ_g_) between adiposity phenotypes, a bivariate LD score regression [57] was conducted on the current GWAS. All of our summary statistics were transformed to a standard format, and LD information was calculated from the 1000 G phase 3 East Asian panel (v2.2). The *p* value of each pair of genetic correlations (*p* value for γ_g_) was then adjusted for the false discovery rate (FDR) for multiple corrections. We further compared our suggested SNP lists with the GWAS catalog to examine pleiotropic effects.

## Supporting information

S1 Fig Schematic showing the flowchart quality control, association analysis and post-GWAS steps in this study. (PDF)

S2 Fig Principle component analysis plots examining population stratification. Substructure for (a) our Taiwanese cohort (TWN) and 1000 Genome and (b) our Taiwanese cohort (TWN) and East Asians. Graphs represent the first two principal components. Different colors illustrate each sub-population. ACB, African Caribbean in Barbados; ASW, African ancestry in Southwest USA; BEB, Bengali in Bangladesh; CDX, Chinese Dai in Xishuangbanna, China; CEU, Utah residents with Northern and Western European ancestry from the CEPH collection; CHB, Han Chinese in Beijing; CHD, Chinese in Metropolitan Denver; CHS, Han Chinese South; CLM, Colombian in Medellin, Colombia; ESN, Esan in Nigeria; FIN, Finnish in Finland; GBR, British in England and Scotland; GIH, Gujarati Indians in Houston; GWD, Gambian in Western Division, The Gambia-Mandinka; IBS, Iberian populations in Spain; ITU, Indian Telugu in the UK; JPT, Japanese in Tokyo; KHV, Kinh in Ho Chi Minh City, Vietnam; LWK, Luhya in Webuye, Kenya; MXL, Mexican Ancestry in Los Angeles, California; MSL, Mende in Sierra Leone; PEL, Peruvian in Lima, Peru; PJL, Punjabi in Lahore, Pakistan; PUR, Puerto Rican in Puerto Rico; STU, Sri Lankan Tamil in the UK; TSI, Toscani in Italia; TWN, Taiwanese; YRI, Yoruba in Ibadan.
(PDF)

S3 Fig Quantile-quantile plots of adiposity phenotypes. BMI, body-mass index; BF% body fat percentage; WC, waist circumference; WHR, waist-hip ratio.
(PDF)

S4 Fig Significant loci of adiposity phenotypes identified in a Taiwanese population. Regional plots before (left) and after (right) a conditional analysis. hg19 of 1000 Genome East Asian version (Nov. 2014) was used as the reference panel. BMI, body-mass index; BF%, body fat percentage; WC, waist circumference.
(PDF)

S5 Fig Functional annotation of extended single-nucleotide polymorphism (SNP) lists of four phenotypes. Functional annotation results from positional mapping by ANNOVAR. The number of extended SNPs of each phenotype is listed in the figure legend. BMI, body-mass index; BF%, body fat percentage; WC, waist circumference; WHR, waist-hip ratio.
(PDF)

S6 Fig Proportions of the open state across different tissue types in the 15-core ChromHMM state model. The figure shows that the proportions of open state (chromHMM state <7) are similar across all tissues. The 127 epigenomes from the 15-core chromatin state model were sorted into 29 tissue categories.
(PDF)

S7 Fig Functional annotations of *RALGAPA1* loci for the four adiposity traits. (PDF)

S8 Fig Subgroup active enhancer enrichment of adiposity variants in (left) connective cells; (middle) fibroblasts; and (right) osteoblasts (“connective and bone” category). The red line denotes *p*<0.05, and the blue line denotes *p*<0.1.
(PDF)

S9 Fig Subgroup active enhancer enrichment of adiposity variants in B cells, T cells, the spleen, and thymus (“immune related” category). The red line denotes *p*<0.05, and the blue line denotes *p*<0.1.
(PDF)

S10 Fig Active enhancer enrichment of neuronal subgroups for the four adiposity traits. (PDF)

S11 Fig Pairwise genetic correlations of four adiposity-related traits. (PDF)

S12 Fig Epigenetic profile of rs8004796 on the UCSC genome browser. A transcriptional factor-binding site, RBM22, was predicted to bind to rs8004796. However, there were no transcriptional activities in the cell lines.
(PDF)

S13 Fig Epigenetic profile of rs76115093 on the UCSC genome browser. rs76115093 showed evidence of histone modification and transcriptional factor binding, and additionally, CTCF modulation was shown in different cell lines. rs76115093 may be a functional variant.
(PDF)

S1 Table Summary statistics of body mass index (BMI) in Taiwanese cohort. (LRZ)

S2 Table Summary statistics of body fat percentage (BF%) in Taiwanese cohort. (LRZ)

S3 Table Summary statistics of waist circumference (WC) in Taiwanese cohort. (LRZ)

S4 Table Summary statistics of waist-hip ratio (WHR) in Taiwanese cohort. (LRZ)

S5 Table Summary of the results of a trans-ethnic meta-analysis. (PDF)

S6 Table Pleiotropy of the 13 adiposity trait-associated loci in a Taiwanese population. (PDF)

S7 Table Frequencies of novel body fat percentage (BF%)-associated single-nucleotide polymorphisms (SNPs) in different populations. (PDF)

S8 Table Single-nucleotide polymorphisms (SNPs) in high linkage disequilibrium (LD) (*r*^*2*^>0.8) to novel body fat percentage (BF%)-associated variants. (PDF)

S9 Table Functional annotations of single-nucleotide polymorphisms (SNPs) using the HaploReg and RegulomeDB databases. (PDF)

S10 Table Comparison of the minimum allele frequency (MAF) of single-nucleotide polymorphisms (SNPs) on *RALGAPA1* (±100-kb region) in 1000G populations. (PDF)
