# Supplementary figures and images for: Genome-wide association study identifies genetic risk loci for adiposity in a Taiwanese population
Source: PLoS Genet. 2022 Jan 20;18(1):e1009952. doi: 10.1371/journal.pgen.1009952 (PMC8853642; doi:10.1371/journal.pgen.1009952)

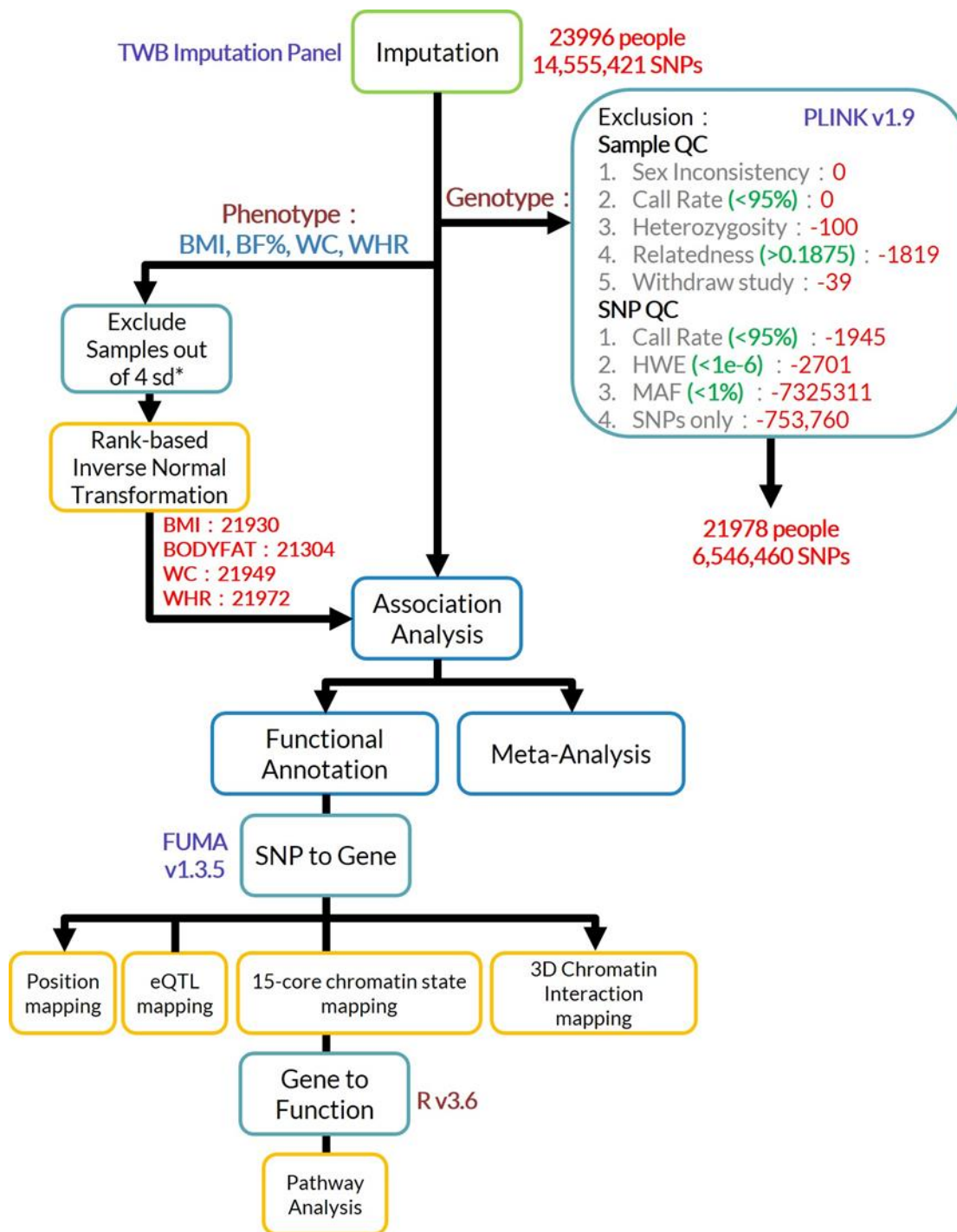

Supplement: S1 Fig — (PDF) [file pgen.1009952.s001.pdf]

a.

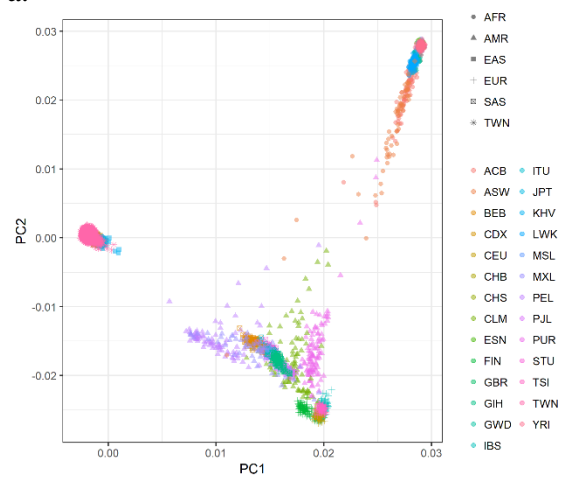

b.

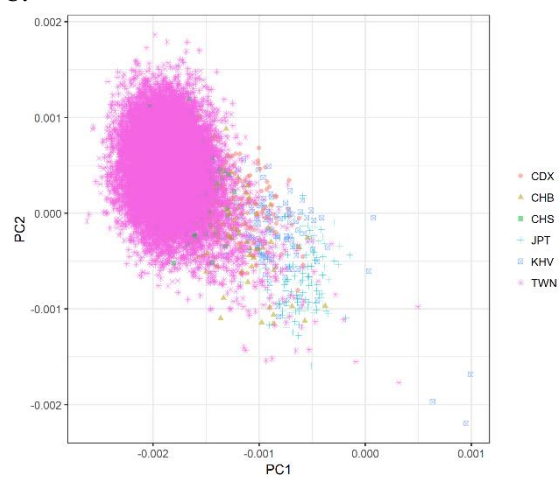

Supplement: S2 Fig — Substructure for (a) our Taiwanese cohort (TWN) and 1000 Genome and (b) our Taiwanese cohort (TWN) and East Asians. Graphs represent the first two principal components. Different colors illustrate each sub-population. ACB, African Caribbean in Barbados; ASW, African ancestry in Southwest USA; BEB, Bengali in Bangladesh; CDX, Chinese Dai in Xishuangbanna, China; CEU, Utah residents with Northern and Western European ancestry from the CEPH collection; CHB, Han Chinese in Beijing; CHD, Chinese in Metropolitan Denver; CHS, Han Chinese South; CLM, Colombian in Medellin, Colombia; ESN, Esan in Nigeria; FIN, Finnish in Finland; GBR, British in England and Scotland; GIH, Gujarati Indians in Houston; GWD, Gambian in Western Division, The Gambia-Mandinka; IBS, Iberian populations in Spain; ITU, Indian Telugu in the UK; JPT, Japanese in Tokyo; KHV, Kinh in Ho Chi Minh City, Vietnam; LWK, Luhya in Webuye, Kenya; MXL, Mexican Ancestry in Los Angeles, California; MSL, Mende in Sierra Leone; PEL, Peruvian in Lima, Peru; PJL, Punjabi in Lahore, Pakistan; PUR, Puerto Rican in Puerto Rico; STU, Sri Lankan Tamil in the UK; TSI, Toscani in Italia; TWN, Taiwanese; YRI, Yoruba in Ibadan. (PDF) [file pgen.1009952.s002.pdf]

a. BMI

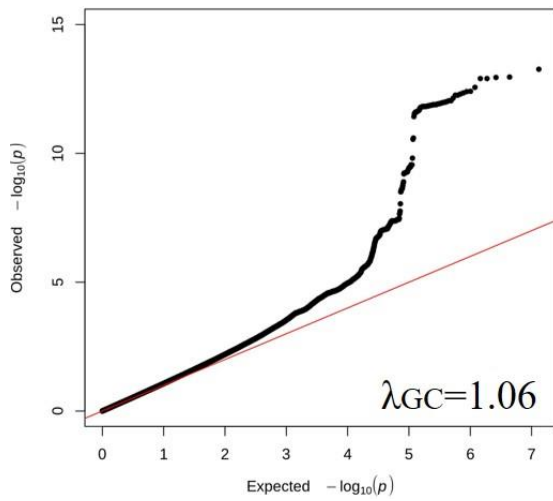

b. BF%

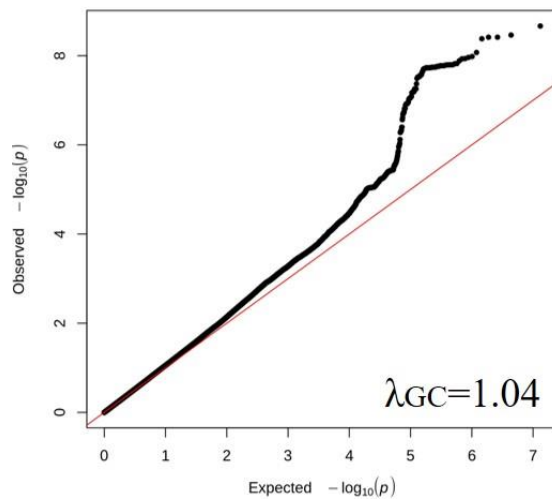

c. WC

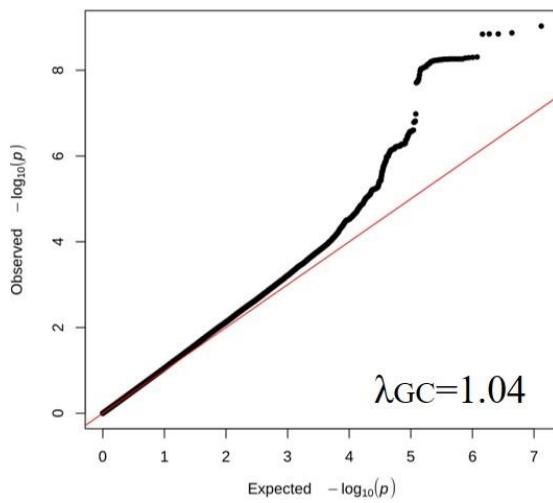

d. WHR

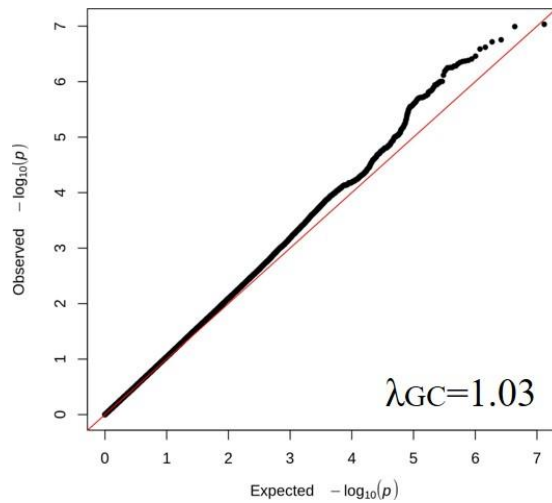

Supplement: S3 Fig — BMI, body-mass index; BF% body fat percentage; WC, waist circumference; WHR, waist-hip ratio. (PDF) [file pgen.1009952.s003.pdf]

a.

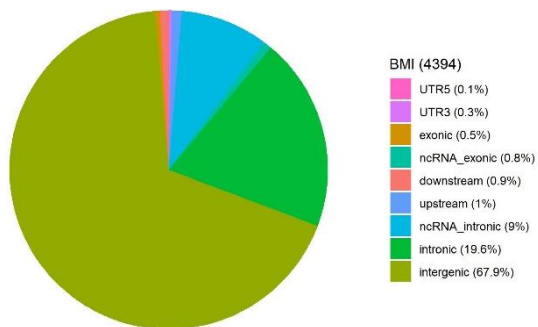

b.

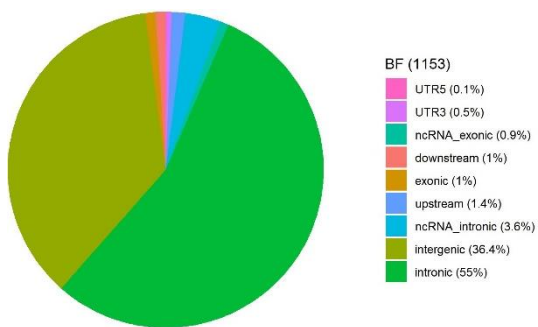

c.

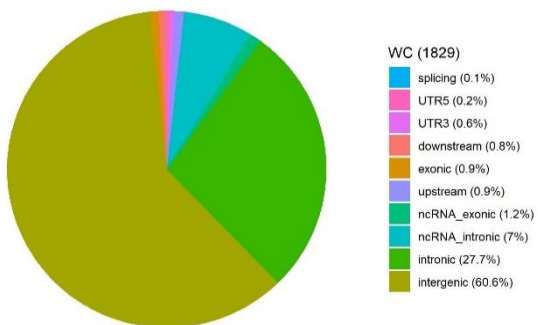

d.

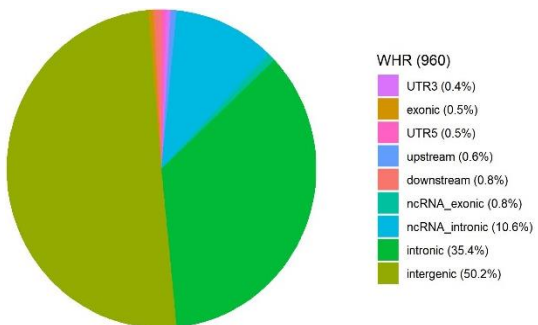

Supplement: S5 Fig — Functional annotation results from positional mapping by ANNOVAR. The number of extended SNPs of each phenotype is listed in the figure legend. BMI, body-mass index; BF%, body fat percentage; WC, waist circumference; WHR, waist-hip ratio. (PDF) [file pgen.1009952.s005.pdf]

### a. BMI

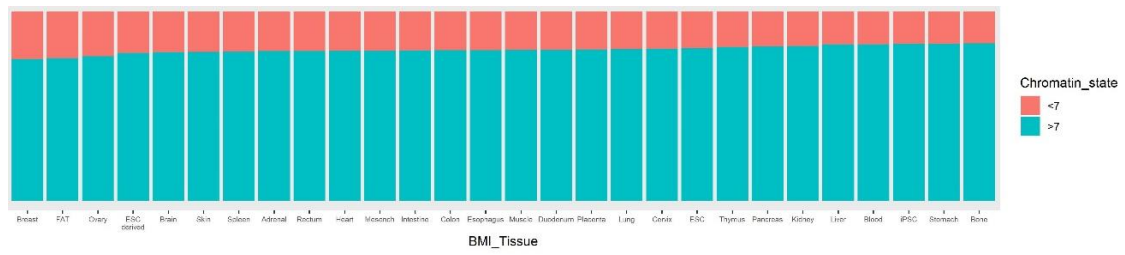

### b. Body fat percentage

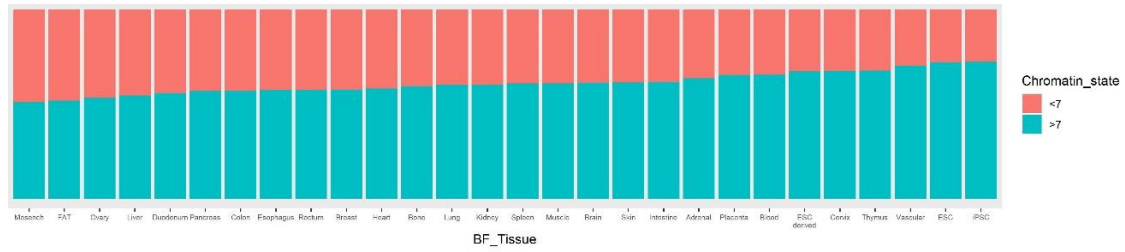

### c. Waist circumference

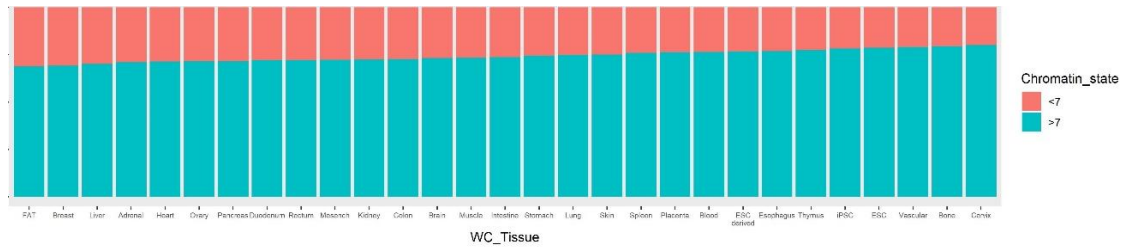

### d. Waist-hip ratio

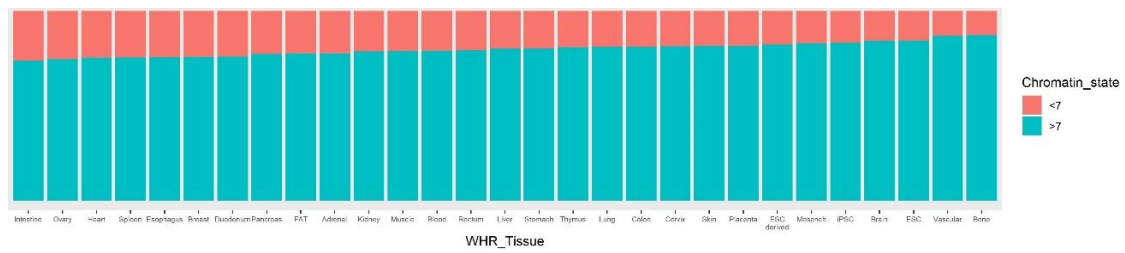

Supplement: S6 Fig — The figure shows that the proportions of open state (chromHMM state <7) are similar across all tissues. The 127 epigenomes from the 15-core chromatin state model were sorted into 29 tissue categories. (PDF) [file pgen.1009952.s006.pdf]

**Connective cells**

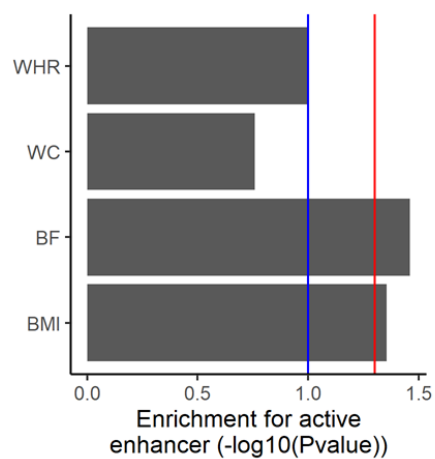

**Fibroblasts**

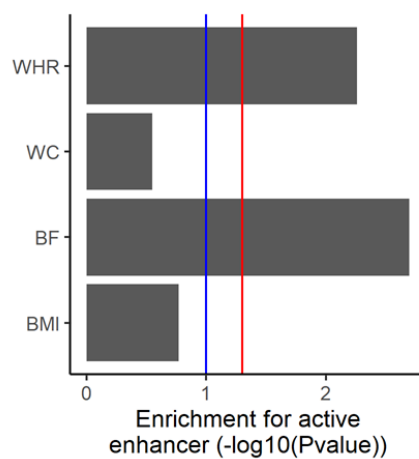

**Osteoblasts**

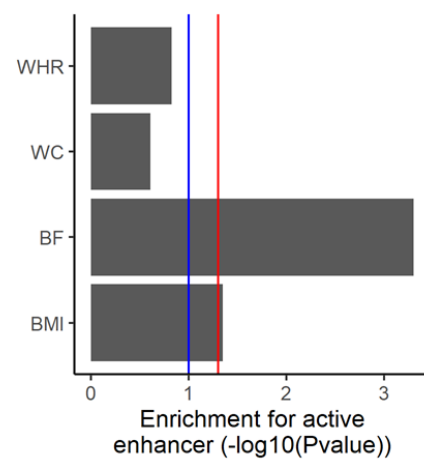

Supplement: S8 Fig — The red line denotes p<0.05, and the blue line denotes p<0.1. (PDF) [file pgen.1009952.s008.pdf]

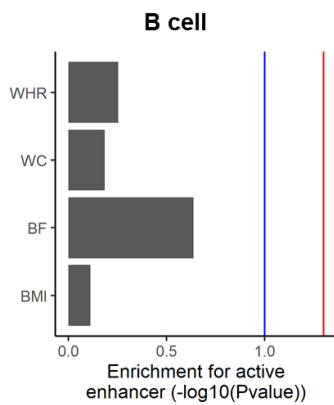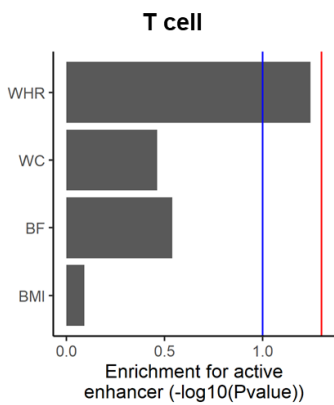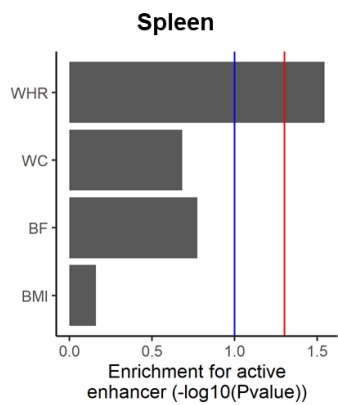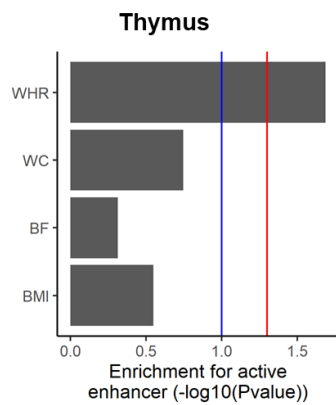

Supplement: S9 Fig — The red line denotes p<0.05, and the blue line denotes p<0.1. (PDF) [file pgen.1009952.s009.pdf]

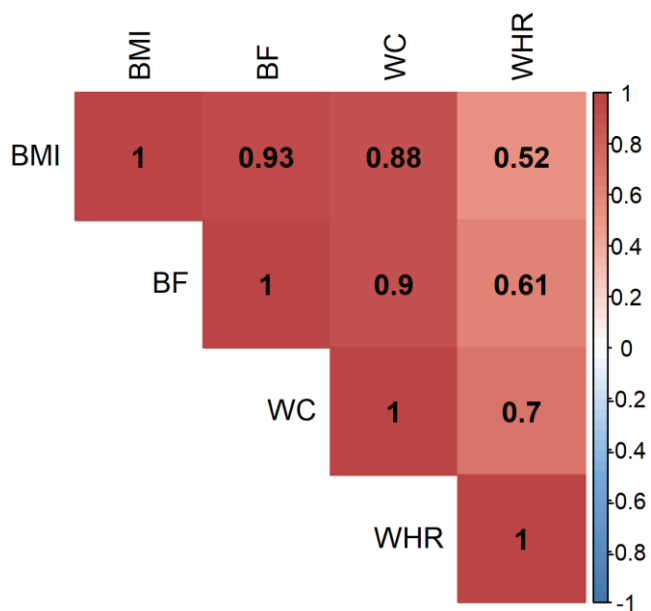

Supplement: S11 Fig — (PDF) [file pgen.1009952.s011.pdf]

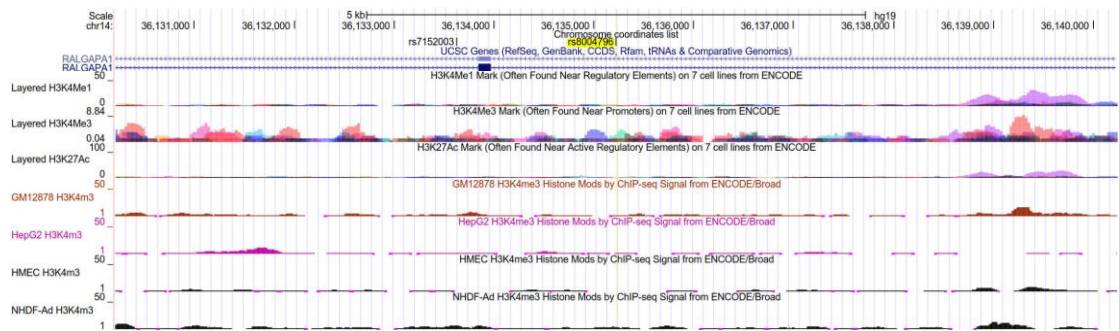

Supplement: S12 Fig — A transcriptional factor-binding site, RBM22, was predicted to bind to rs8004796. However, there were no transcriptional activities in the cell lines. (PDF) [file pgen.1009952.s012.pdf]

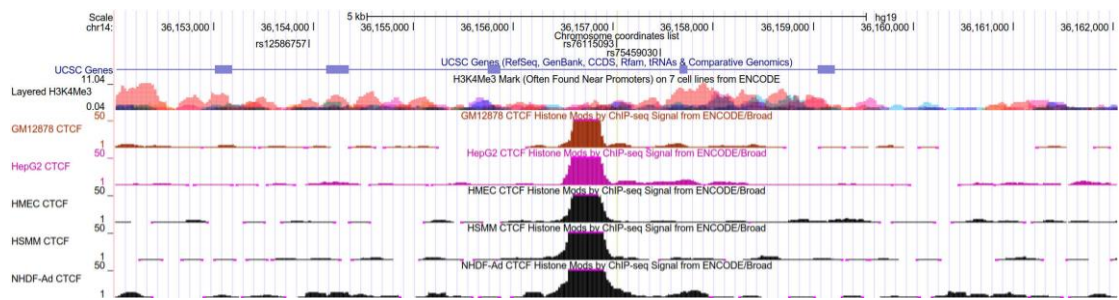

Supplement: S13 Fig — rs76115093 showed evidence of histone modification and transcriptional factor binding, and additionally, CTCF modulation was shown in different cell lines. rs76115093 may be a functional variant. (PDF) [file pgen.1009952.s013.pdf]
