# Supplementary material for: Genome-wide association study identifies genetic risk loci for adiposity in a Taiwanese population
Source: PLoS Genet. 2022 Jan 20;18(1):e1009952. doi: 10.1371/journal.pgen.1009952 (PMC8853642; doi:10.1371/journal.pgen.1009952)
Supplement: S4 Fig — Regional plots before (left) and after (right) a conditional analysis. hg19 of 1000 Genome East Asian version (Nov. 2014) was used as the reference panel. BMI, body-mass index; BF%, body fat percentage; WC, waist circumference. (PDF) [file pgen.1009952.s004.pdf]

BMI

chr4: 44175691−46175691

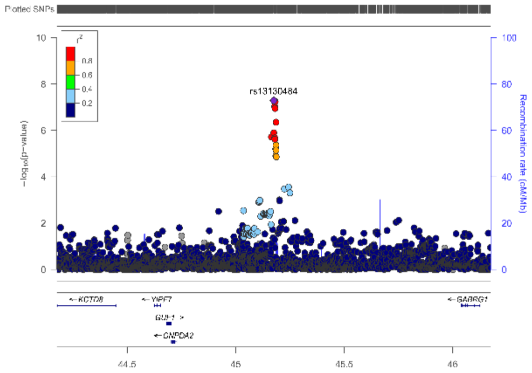

chr4: 44175691−46175691

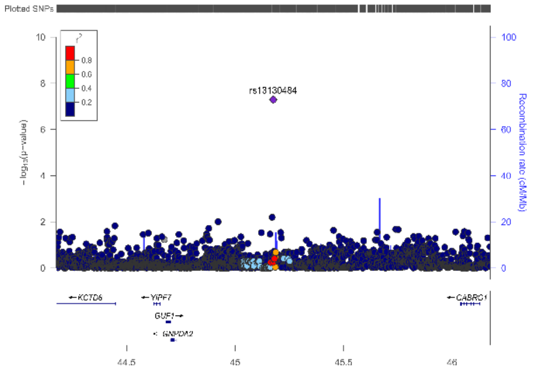

chr6: 49855795−51855795

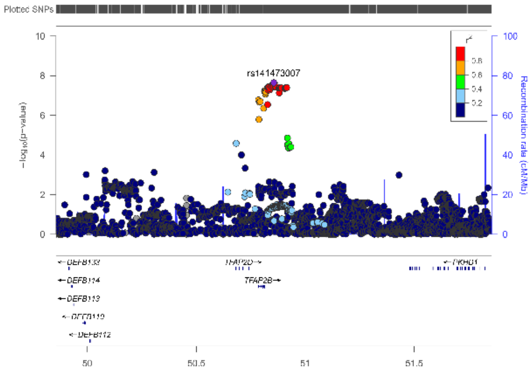

chr6: 49855795−51855795

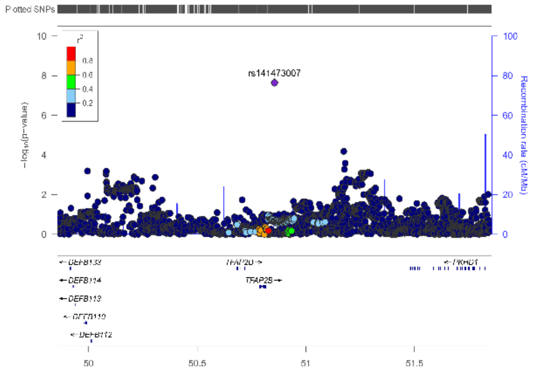

chr14 :35135219−37135219

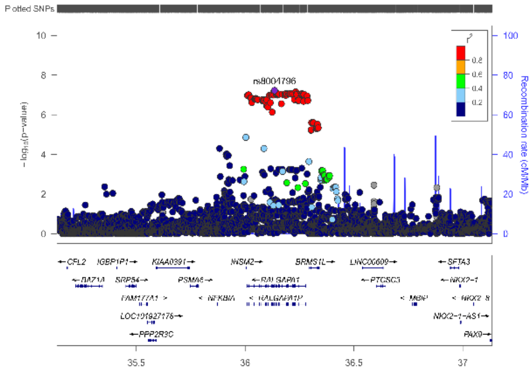

chr14 :35135219−37135219

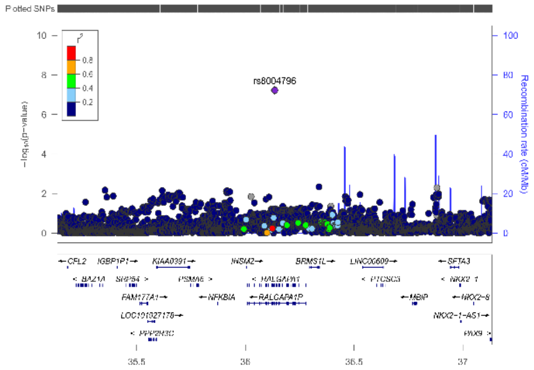

chr16: 52800954−54800954

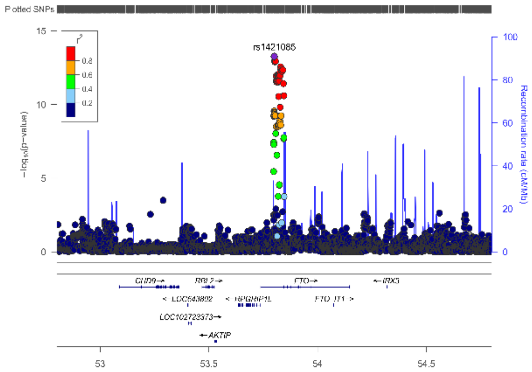

chr16: 52800954−54800954

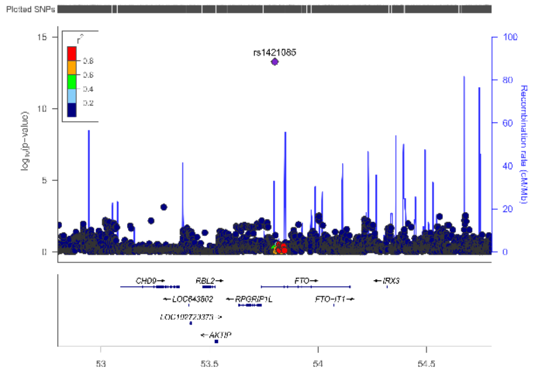

BMI

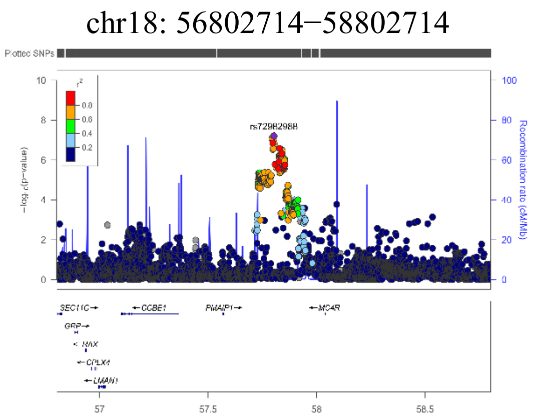
chr18: 56802714−58802714
Plotted SNPs
rs72982988
SEC11C
CCBE1
PMAIP1
MC4R
GRP
RAX
CPLX4
LMAN1
57
57.5
58
58.5
Recombination rate (cM/Mb)

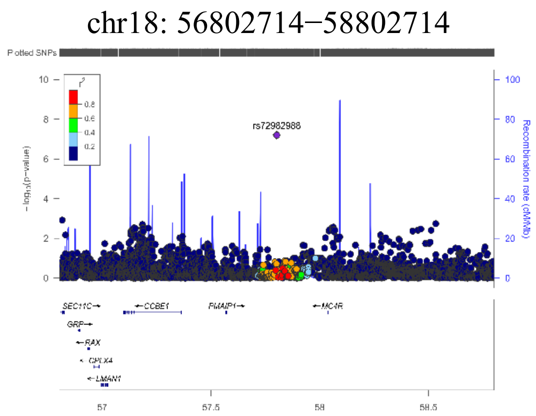
chr18: 56802714−58802714
Plotted SNPs
rs72982988
SEC11C
CCBE1
PMAIP1
MC4R
GRP
RAX
CPLX4
LMAN1
57
57.5
58
58.5
Recombination rate (cM/Mb)

BF%

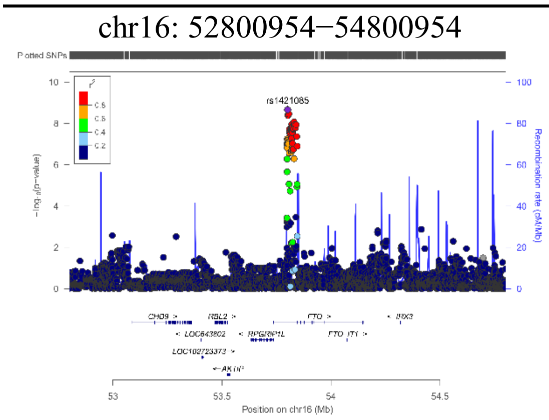
chr16: 52800954−54800954
Plotted SNPs
rs1421085
CHD9
RBL2
FTO
IRX3
LOC643802
RPGRIP1L
FTO-IT1
LOC102723373
AKTIP
53
53.5
54
54.5
Position on chr16 (Mb)
Recombination rate (cM/Mb)

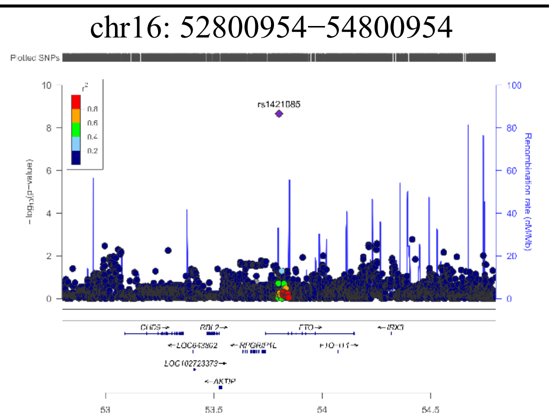
chr16: 52800954−54800954
Plotted SNPs
rs1421085
CHD9
RBL2
FTO
IRX3
LOC643802
RPGRIP1L
FTO-IT1
LOC102723373
AKTIP
53
53.5
54
54.5
Recombination rate (cM/Mb)

WC

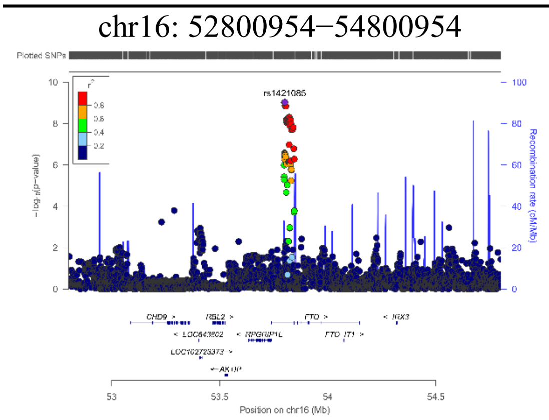
chr16: 52800954−54800954
Plotted SNPs
rs1421085
CHD9
RBL2
FTO
IRX3
LOC643802
RPGRIP1L
FTO-IT1
LOC102723373
AKTIP
53
53.5
54
54.5
Position on chr16 (Mb)
Recombination rate (cM/Mb)

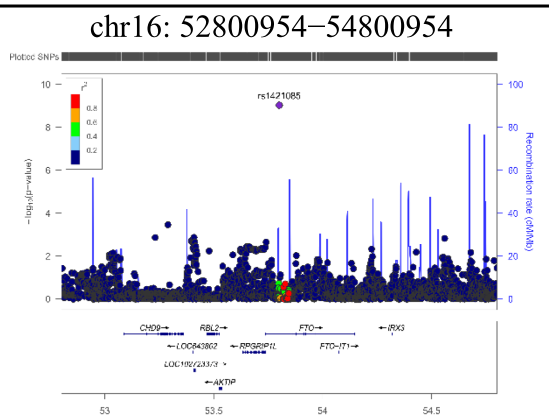
chr16: 52800954−54800954
Plotted SNPs
rs1421085
CHD9
RBL2
FTO
IRX3
LOC643802
RPGRIP1L
FTO-IT1
LOC102723373
AKTIP
53
53.5
54
54.5
Recombination rate (cM/Mb)
