# Supplementary material for: Genome-wide association study identifies genetic risk loci for adiposity in a Taiwanese population
Source: PLoS Genet. 2022 Jan 20;18(1):e1009952. doi: 10.1371/journal.pgen.1009952 (PMC8853642; doi:10.1371/journal.pgen.1009952)
Supplement: S7 Fig — (PDF) [file pgen.1009952.s007.pdf]

Trait	CADD score >12.37	Chromatin state mapping based on 15-core ChromHMM model	3D chromatin interaction mapping based on Hi-C data (GSE87112) of 21 tissues/cell types
BMI	Yes	Yes	Yes
BF%	Yes	Yes	Yes
WC	Yes	Yes	Yes
WHR	No	No	No
