# Supplementary material for: Genome-wide association study identifies genetic risk loci for adiposity in a Taiwanese population
Source: PLoS Genet. 2022 Jan 20;18(1):e1009952. doi: 10.1371/journal.pgen.1009952 (PMC8853642; doi:10.1371/journal.pgen.1009952)
Supplement: S10 Fig — (PDF) [file pgen.1009952.s010.pdf]

phenotype	tissue	fdr
BMI	Hippocampus_Middle	0.09975
BMI	Substantia_Nigra	0.4135
BMI	Anterior_Caudate	0.089833333
BMI	Cingulate_Gyrus	0.089833333
BMI	Inferior_Temporal_Lobe	0.089833333
BMI	Angular_Gyrus	0.2282
BMI	Mid_Frontal_Lobe	0.4135
BF%	Hippocampus_Middle	0.0917
BF%	Substantia_Nigra	0.228
BF%	Anterior_Caudate	0.049
BF%	Cingulate_Gyrus	0.021
BF%	Inferior_Temporal_Lobe	0.021
BF%	Angular_Gyrus	0.0917
BF%	Mid_Frontal_Lobe	0.228
WC	Hippocampus_Middle	0.1505
WC	Substantia_Nigra	0.1925
WC	Anterior_Caudate	0.20475
WC	Cingulate_Gyrus	0.2715
WC	Inferior_Temporal_Lobe	0.2715
WC	Angular_Gyrus	0.2715
WC	Mid_Frontal_Lobe	0.1925
WHR	Hippocampus_Middle	0.154
WHR	Substantia_Nigra	0.154
WHR	Anterior_Caudate	0.154
WHR	Cingulate_Gyrus	0.5245
WHR	Inferior_Temporal_Lobe	0.5245
WHR	Angular_Gyrus	0.5245
WHR	Mid_Frontal_Lobe	0.30275
