# Supplementary material for: Genome-wide association study identifies genetic risk loci for adiposity in a Taiwanese population
Source: PLoS Genet. 2022 Jan 20;18(1):e1009952. doi: 10.1371/journal.pgen.1009952 (PMC8853642; doi:10.1371/journal.pgen.1009952)
Supplement: S5 Table — (PDF) [file pgen.1009952.s018.pdf]

S5 Table. Summary of the results of a trans-ethnic meta-analysis

Phenotype	Meta-analysis	GWS	GWS AL	Availability 1	GWS_NomSig&SameDir	SS	Availability 2	SS Replication	SS AL
BMI	TWB+UK	130	2	130 (100%)	130 (100%)	268	268 (100%)	183 (68.28%)	5
BMI	TWB+BBJ			130 (100%)	118 (90.77%)	268	268 (100%)	250 (93.28%)	6
BMI	TWB+GERA			130 (100%)	118 (90.77%)	268	268 (100%)	244 (91.04%)	7
BF%	TWB+UK	53	1	53 (100%)	53 (100%)	100	100 (100%)	94 (94.00%)	3
BF%	TWB+Hübel			53 (100%)	26 (49.06%)	100	100 (100%)	50 (50.00%)	3
WC	TWB+UK	54	1	54 (100%)	54 (100%)	152	152 (100%)	91 (59.87%)	2
WC	TWB+GIANT			54 (100%)	17 (31.48%)	152	152 (100%)	73 (48.03%)	2
WHR	TWB+GIANT	0	n.a.	n.a.	n.a.	25	25 (100%)	3 (12.00%)	1

GWS, no. of single-nucleotide polymorphisms (SNPs) that reached genome-wide sequence significance (GWS) (p<5E-8). GWS UVL, no. of associated loci (ALs) of GWS SNPs (that were at least 1 Mb apart). Availability 1, the no. (and percentage) of available GWS SNPs (from the Taiwan Biobank (TWB)) in the replication set. GWS NomSig&SameDir, the no. (and percentage) of available GWS SNPs that reached nominal significance (nominal p<0.05) in the meta-analysis and had the same effect direction as in the replication set; SS, no. of SNPs that reached the suggested significant threshold (p<1E-6). Availability 2, the no. (and percentage) of available SS SNPs (from the TWB) in the replication set. SS Replication, the no. (and percentage) of SNPs that reached GWS in the meta-analysis (*i.e.*, were successfully replicated). SS AL, the no. of ALs of the replicated SNPs (which were at least 1 Mb apart). n.a., not applicable.
