# Supplementary material for: Genome-wide association study identifies genetic risk loci for adiposity in a Taiwanese population
Source: PLoS Genet. 2022 Jan 20;18(1):e1009952. doi: 10.1371/journal.pgen.1009952 (PMC8853642; doi:10.1371/journal.pgen.1009952)
Supplement: S6 Table — (PDF) [file pgen.1009952.s019.pdf]

S6 Table. Pleiotropy of the 13 adiposity trait-associated loci in a Taiwanese population

Representative SNP [a]	Associated traits in this study	No.	Traits according to the genome-wide association study (GWAS) catalog
rs6567160	BMI	6	Obesity, height, high-density lipoprotein (HDL) cholesterol, obesity (early-onset extreme), body-mass index (BMI), fat body mass
rs10938397	BMI, BF%	3	BMI, obesity, menarche (age at onset)
rs11642015	BF%, WC	19	BMI, obesity (early-onset extreme), HDL cholesterol, triglycerides, obesity, dietary macronutrient intake, waist circumference, height-adjusted BMI, breast cancer, type 2 diabetes, weight, body mass in chronic obstructive pulmonary disease, adiposity, menarche (age at onset), subcutaneous adipose tissue, obesity-related traits, biomedical quantitative traits, metabolic syndrome
rs1558902	BMI	19	BMI, obesity (early-onset extreme), HDL cholesterol, triglycerides, obesity, dietary macronutrient intake, waist circumference, height-adjusted BMI, breast cancer, type 2 diabetes, weight, body mass in chronic obstructive pulmonary disease, adiposity, menarche (age at onset), subcutaneous adipose tissue, obesity-related traits, biomedical quantitative traits, metabolic syndrome, obesity (extreme)

[a] To detect the pleiotropic effects of the 13 loci (associated with one of the adiposity traits), each represented single-nucleotide polymorphism (SNP) (with the highest *p* value) was expanded by the linkage disequilibrium (LD) threshold of r^2>0.9, and then the represented SNP (the query SNP) and the expanded SNPs were queried for reported associations for other traits. The reported traits were then assigned to the representative SNP.
