# Supplementary material for: Genome-wide association study identifies genetic risk loci for adiposity in a Taiwanese population
Source: PLoS Genet. 2022 Jan 20;18(1):e1009952. doi: 10.1371/journal.pgen.1009952 (PMC8853642; doi:10.1371/journal.pgen.1009952)
Supplement: S7 Table — (PDF) [file pgen.1009952.s020.pdf]

**S7 Table.** Frequencies of novel body fat percentage (BF%)-associated single-nucleotide polymorphisms (SNPs) in different populations

| SNP | Chr:pos [hg19] | RA[a] | AF [b] of RA in 1KGP [c] | | | AF [b] of RA in gnomAD – Genomes [d] | | | TWB [e] |
|---|---|---|---|---|---|---|---|---|---|
| | | | European | African | East Asian | European | African | East Asian | |
| rs10938397 | 4:45182527 | A | 0.580 | 0.796 | 0.697 | 0.561 | 0.752 | 0.712 | 0.731 |
| rs28376697 | 15:68140315 | G | 0.226 | 0.034 | 0.544 | 0.188 | 0.056 | 0.553 | 0.473 |

[a] Risk allele. [b] Allele frequency. [c] 1000 Genomes project. [d] The genome Aggregation Database. [e] Taiwanese Biobank project.
