# Supplementary material for: Genome-wide association study identifies genetic risk loci for adiposity in a Taiwanese population
Source: PLoS Genet. 2022 Jan 20;18(1):e1009952. doi: 10.1371/journal.pgen.1009952 (PMC8853642; doi:10.1371/journal.pgen.1009952)
Supplement: S8 Table — (PDF) [file pgen.1009952.s021.pdf]

S8 Table. Single-nucleotide polymorphisms (SNPs) in high linkage disequilibrium (LD) (r^2>0.8) to novel body fat percentage (BF%)-associated variants

CHR_A	BP_A	SNP_A	CHR_B	BP_B	SNP_B	R^2	Func.refGene	Gene.refGene	cytoBand	MAF
4	45182527	rs10938397	4	45164637	rs1996023	0.862317	intergenic	*GNPDA2-GABRG1*	4p12	0.2721
4	45182527	rs10938397	4	45175691	rs13130484	0.999857	intergenic	*GNPDA2-GABRG1*	4p12	0.2636
4	45182527	rs10938397	4	45175804	rs16858082	0.864598	intergenic	*GNPDA2-GABRG1*	4p12	0.2933
4	45182527	rs10938397	4	45179883	rs12641981	0.994551	intergenic	*GNPDA2-GABRG1*	4p12	0.2623
4	45182527	rs10938397	4	45180197	rs1581095	0.859562	intergenic	*GNPDA2-GABRG1*	4p12	0.292
4	45182527	rs10938397	4	45180999	rs16858086	0.859845	intergenic	*GNPDA2-GABRG1*	4p12	0.2921
4	45182527	rs10938397	4	45181334	rs12507026	0.99498	intergenic	*GNPDA2-GABRG1*	4p12	0.2625
4	45182527	rs10938397	4	45182527	rs10938397	1	intergenic	*GNPDA2-GABRG1*	4p12	0.2638
4	45182527	rs10938397	4	45184442	rs348495	0.846446	intergenic	*GNPDA2-GABRG1*	4p12	0.2934
4	45182527	rs10938397	4	45185876	rs2062579	0.855871	intergenic	*GNPDA2-GABRG1*	4p12	0.2918
4	45182527	rs10938397	4	45186139	rs10938398	0.9878	intergenic	*GNPDA2-GABRG1*	4p12	0.261
4	45182527	rs10938397	4	45186253	rs348492	0.853095	intergenic	*GNPDA2-GABRG1*	4p12	0.2908
4	45182527	rs10938397	4	45186832	rs348500	0.849718	intergenic	*GNPDA2-GABRG1*	4p12	0.2884
15	68140315	rs28376697	15	68137364	rs4776990	0.973817	intergenic	*RNU6-2-PIAS1*	15q23	0.4439
15	68140315	rs28376697	15	68140315	rs28376697	1	intergenic	*RNU6-2-PIAS1*	15q23	0.4419
15	68140315	rs28376697	15	68140604	rs12592195	0.836925	intergenic	*RNU6-2-PIAS1*	15q23	0.3981
15	68140315	rs28376697	15	68147419	rs12148694	0.81018	intergenic	*RNU6-2-PIAS1*	15q23	0.3965
15	68140315	rs28376697	15	68147425	rs12148234	0.81018	intergenic	*RNU6-2-PIAS1*	15q23	0.3965

CHR_A, chromosome of SNP_A; BP_A, base pair position of SNP_A; SNP_A, query SNP (novel BF%-associated SNP); BP_B, chromosome of SNP_B, BP_B, base pair position of SNP_B; SNP_B, SNP in high LD to the query SNP; R^2, r^2 value between SNP_A and SNP_B; MAF, minor allele frequency of the SNP_B based on Taiwan Biobank project.
