# Supplementary material for: Genome-wide association study identifies genetic risk loci for adiposity in a Taiwanese population
Source: PLoS Genet. 2022 Jan 20;18(1):e1009952. doi: 10.1371/journal.pgen.1009952 (PMC8853642; doi:10.1371/journal.pgen.1009952)
Supplement: S9 Table — (PDF) [file pgen.1009952.s022.pdf]

S9 Table. Functional annotations of single-nucleotide polymorphisms (SNPs) using the HaploReg and RegulomeDB databases

SNP	CS	CS_I	CM	DNAse	Proteins	eQTL	grasp	Motifs	FA	PHM	EHM	Sum	Avg.
rs10938397	0	0	1	0	0	0	1	1	0	0	0	3	0.272727
rs10938398	0	0	1	0	0	0	0	1	0	0	0	2	0.181818
rs12507026	0	1	1	1	1	0	0	1	0	0	0	5	0.454545
rs12641981	0	0	1	0	0	0	1	1	0	0	0	3	0.272727
rs13130484	0	0	1	0	0	0	1	1	0	0	0	3	0.272727
rs1581095	1	0	1	0	0	0	0	1	0	0	1	4	0.363636
rs16858082	0	0	1	0	0	0	0	0	0	0	0	1	0.090909
rs16858086	0	0	1	0	0	0	0	1	0	0	0	2	0.181818
rs1996023	0	0	0	0	0	0	0	1	0	0	0	1	0.090909
rs2062579	1	1	1	0	0	0	0	1	0	0	1	5	0.454545
rs348492	1	0	1	0	0	0	0	1	0	0	1	4	0.363636
rs348495	0	0	1	0	0	0	0	1	0	0	0	2	0.181818
rs348500	1	0	1	0	0	0	0	1	0	0	1	4	0.363636
rs12148234	1	0	1	0	0	1	0	1	0	0	1	5	0.454545
rs12148694	1	0	1	0	0	1	0	1	0	0	1	5	0.454545
rs12592195	1	1	1	1	0	1	0	1	0	0	1	7	0.636364
rs28376697	1	1	1	1	0	1	0	1	0	0	1	7	0.636364
rs4776990	1	1	1	1	0	1	0	0	0	0	1	6	0.545455

0, no relevant annotation(s); 1, with available relevant annotation(s); CS, chromatin state; CS_I, chromatin state based on imputed data; CM, chromatin mark; eQTL, expression quantitative trait loci; FA, annotated protein based on the SNP (dbSNP); PHM, promoter histone mark; EHM, enhancer histone mark. Sum, no. of available functional annotation(s); Avg., mean of available functional annotation(s).
