# Supplementary material for: Genome-wide association study identifies genetic risk loci for adiposity in a Taiwanese population
Source: PLoS Genet. 2022 Jan 20;18(1):e1009952. doi: 10.1371/journal.pgen.1009952 (PMC8853642; doi:10.1371/journal.pgen.1009952)
Supplement: S10 Table — (PDF) [file pgen.1009952.s023.pdf]

S10 Table. Comparison of the minimum allele frequency (MAF) of single-nucleotide polymorphisms (SNPs) on *RALGAPA1* (±100-kb region) in 1000G populations

rsID	REF	ALT	BETA	SE	*p*	Position in gene	Candidate gene	cytoBand	TWB_MAF	EAS_MAF	AMR_MAF	AFR_MAF	EUR_MAF	SAS_MAF
rs8004796	C	T	-0.101	0.019	5.96E-08	intronic	RALGAPA1	14q13.2	0.07	0.063	0.016	0.134	0.001	0.175
rs79916424	T	C	-0.1	0.019	6.93E-08	intronic	RALGAPA1	14q13.2	0.07	0.063	0.007	0.056	0.001	0.175
rs12587992	T	C	-0.1	0.019	8.20E-08	intronic	RALGAPA1	14q13.2	0.07	0.063	0.007	0.058	0.001	0.189
rs75626973	A	C	-0.099	0.019	8.54E-08	intronic	RALGAPA1	14q13.2	0.07	0.063	0.016	0.137	0.001	0.176
rs149168276	C	T	-0.099	0.019	8.54E-08	intronic	RALGAPA1	14q13.2	0.07	0.063	0.007	0.063	0.001	0.176
rs58804067	G	C	-0.099	0.019	8.54E-08	intronic	RALGAPA1	14q13.2	0.07	0.063	0.007	0.062	0.001	0.178
rs59505918	T	A	-0.099	0.019	8.54E-08	intronic	RALGAPA1	14q13.2	0.07	0.063	0.007	0.061	0.001	0.176
rs12588266	T	G	-0.099	0.019	8.62E-08	intronic	RALGAPA1	14q13.2	0.07	0.064	0.007	0.058	0.001	0.177
rs61613627	C	T	-0.103	0.019	8.65E-08	intronic	RALGAPA1	14q13.2	0.066	0.063	0.007	0.057	0.001	0.176
rs12590376	A	G	-0.099	0.019	8.72E-08	intronic	RALGAPA1	14q13.2	0.07	0.063	0.007	0.06	0.001	0.176
rs79111861	G	T	-0.099	0.019	8.72E-08	intronic	RALGAPA1	14q13.2	0.07	0.063	0.007	0.055	0.001	0.176
rs185108225	T	C	-0.099	0.019	8.72E-08	intronic	RALGAPA1	14q13.2	0.07	0.063	0.007	0.058	0.001	0.176
rs12587686	C	T	-0.099	0.019	8.72E-08	exonic	RALGAPA1	14q13.2	0.07	0.063	0.007	0.058	0.001	0.176
rs114818991	A	G	-0.099	0.019	8.72E-08	intronic	RALGAPA1	14q13.2	0.07	0.063	0.007	0.056	0.001	0.176
rs12586147	G	A	-0.099	0.019	8.93E-08	intronic	RALGAPA1	14q13.2	0.07	0.063	0.007	0.055	0.001	0.177
rs115172438	T	C	-0.099	0.019	8.95E-08	intronic	RALGAPA1	14q13.2	0.07	0.063	0.007	0.063	0.001	0.176
rs12587569	C	T	-0.099	0.019	8.95E-08	intronic	RALGAPA1	14q13.2	0.07	0.063	0.007	0.058	0.001	0.176
rs78129608	T	A	-0.099	0.019	9.15E-08	intronic	RALGAPA1	14q13.2	0.07	0.063	0.007	0.017	0.001	0.177
rs12586757	G	A	-0.099	0.019	9.15E-08	intronic	RALGAPA1	14q13.2	0.07	0.063	0.007	0.017	0.001	0.176
rs76115093	T	C	-0.099	0.019	9.15E-08	intronic	RALGAPA1	14q13.2	0.07	0.063	0.007	0.017	0.001	0.176
rs75459030	T	C	-0.099	0.019	9.15E-08	intronic	RALGAPA1	14q13.2	0.07	0.063	0.007	0.06	0.001	0.176
rs116218007	G	A	-0.099	0.019	9.18E-08	intronic	RALGAPA1	14q13.2	0.07	0.063	0.007	0.061	0.003	0.177
rs75871822	T	C	-0.099	0.019	9.18E-08	intronic	RALGAPA1	14q13.2	0.07	0.063	0.016	0.137	0.001	0.176
rs8019535	A	G	-0.099	0.019	9.22E-08	intronic	RALGAPA1	14q13.2	0.07	0.063	0.026	0.205	0.001	0.177
rs145629533	G	A	-0.099	0.019	9.27E-08	intronic	RALGAPA1	14q13.2	0.07	0.063	0.007	0.056	0.001	0.176
rs114462771	C	A	-0.099	0.019	9.50E-08	intronic	RALGAPA1	14q13.2	0.07	0.063	0.007	0.056	0.001	0.176
rs116430039	T	C	-0.099	0.019	9.53E-08	intronic	RALGAPA1	14q13.2	0.07	0.063	0.007	0.058	0.001	0.176
rs12587024	C	T	-0.099	0.019	9.53E-08	intronic	RALGAPA1	14q13.2	0.07	0.063	0.007	0.055	0.001	0.176
rs147946388	T	G	-0.099	0.019	9.64E-08	intronic	RALGAPA1	14q13.2	0.07	0.064	0.007	0.059	0.001	0.177
rs59891257	T	C	-0.099	0.019	9.78E-08	intronic	RALGAPA1	14q13.2	0.07	0.067	0.013	0.067	0.001	0.184
rs78380333	T	C	-0.099	0.019	9.82E-08	intronic	RALGAPA1	14q13.2	0.07	0.063	0.007	0.063	0.001	0.176
rs7152003	A	G	-0.099	0.019	9.86E-08	intronic	RALGAPA1	14q13.2	0.07	0.063	0.016	0.136	0.001	0.178
rs146492551	G	C	-0.099	0.019	9.86E-08	intronic	RALGAPA1	14q13.2	0.07	0.063	0.007	0.058	0.001	0.177
rs114590156	C	T	-0.099	0.019	9.93E-08	intronic	RALGAPA1	14q13.2	0.07	0.063	0.016	0.132	0.001	0.174
rs114357853	A	G	-0.099	0.019	9.93E-08	intronic	RALGAPA1	14q13.2	0.07	0.063	0.016	0.133	0.001	0.176
rs116994836	C	T	-0.099	0.019	9.99E-08	intronic	RALGAPA1	14q13.2	0.07	0.063	0.004	0.002	0.002	0.175
rs75308477	A	C	-0.099	0.019	1.00E-07	intronic	RALGAPA1	14q13.2	0.07	0.063	0.007	0.059	0.001	0.177
rs146287306	T	C	-0.1	0.019	1.01E-07	intronic	RALGAPA1	14q13.2	0.069	0.065	0.009	0.056	0.001	0.176
rs8021912	G	A	-0.099	0.019	1.04E-07	intronic	RALGAPA1	14q13.2	0.07	0.063	0.01	0.156	0.001	0.176
rs115549549	T	C	-0.099	0.019	1.07E-07	intronic	RALGAPA1	14q13.2	0.07	0.067	0.01	0.058	0.001	0.184
rs74943345	T	C	-0.099	0.019	1.07E-07	intronic	RALGAPA1	14q13.2	0.07	0.067	0.01	0.057	0.001	0.184

rs60232901	C	T	-0.099	0.019	1.12E-07	intergenic	RALGAPA1, BRMS1L	14q13.2	0.07	0.063	0.007	0.057	0.001	0.176
rs76876304	T	A	-0.098	0.019	1.42E-07	intronic	RALGAPA1	14q13.2	0.07	0.066	0.01	0.057	0.001	0.183
rs12589742	G	A	-0.098	0.019	1.42E-07	intronic	RALGAPA1	14q13.2	0.07	0.066	0.01	0.058	0.001	0.183
rs3818573	C	T	-0.098	0.019	1.50E-07	intronic	RALGAPA1	14q13.2	0.07	0.065	0.017	0.131	0.001	0.176
rs76178116	G	T	-0.098	0.019	1.50E-07	intronic	RALGAPA1	14q13.2	0.07	0.065	0.009	0.058	0.001	0.176
rs77598147	T	G	-0.097	0.019	1.63E-07	intronic	RALGAPA1	14q13.2	0.07	0.065	0.009	0.058	0.001	0.176
rs75633890	T	C	-0.097	0.019	1.63E-07	intronic	RALGAPA1	14q13.2	0.07	0.065	0.009	0.059	0.001	0.176
rs75059852	A	G	-0.097	0.019	1.63E-07	intronic	RALGAPA1	14q13.2	0.07	0.065	0.009	0.059	0.002	0.177
rs7155743	C	T	-0.097	0.019	1.63E-07	intronic	RALGAPA1	14q13.2	0.07	0.065	0.017	0.131	0.001	0.176
rs114627661	A	G	-0.097	0.019	1.63E-07	intronic	RALGAPA1	14q13.2	0.07	0.065	0.009	0.059	0.001	0.177
rs75172465	C	T	-0.097	0.019	1.68E-07	intronic	RALGAPA1	14q13.2	0.07	0.065	0.017	0.131	0.001	0.176
rs7152755	T	C	-0.097	0.019	1.71E-07	intronic	RALGAPA1	14q13.2	0.07	0.065	0.017	0.131	0.001	0.177
rs74358553	C	T	-0.097	0.019	1.72E-07	intronic	RALGAPA1	14q13.2	0.07	0.065	0.006	0.002	0.001	0.176
rs118026169	C	T	-0.097	0.019	1.72E-07	intronic	RALGAPA1	14q13.2	0.07	0.065	0.006	0.002	0.001	0.174
rs114629850	T	A	-0.097	0.019	1.72E-07	intronic	RALGAPA1	14q13.2	0.07	0.063	0.007	0.058	0.001	0.176
rs114011667	G	A	-0.097	0.019	1.74E-07	intronic	RALGAPA1	14q13.2	0.07	0.065	0.009	0.058	0.001	0.176
rs12589884	T	C	-0.097	0.019	1.87E-07	intronic	RALGAPA1	14q13.2	0.07	0.063	0.007	0.058	0.001	0.176
rs75412345	C	T	-0.097	0.019	1.87E-07	intronic	RALGAPA1	14q13.2	0.07	0.063	0.007	0.058	0.001	0.176
rs114478816	C	T	-0.097	0.019	1.87E-07	intronic	RALGAPA1	14q13.2	0.07	0.063	0.007	0.058	0.001	0.176
rs78041608	A	C	-0.097	0.019	1.89E-07	intronic	RALGAPA1	14q13.2	0.07	0.065	0.009	0.059	0.001	0.177
rs140666599	T	C	-0.097	0.019	1.89E-07	intergenic	RALGAPA1, BRMS1L	14q13.2	0.07	0.063	0.007	0.059	0.001	0.178
rs12588288	C	A	-0.097	0.019	1.97E-07	intronic	RALGAPA1	14q13.2	0.07	0.065	0.009	0.059	0.001	0.176
rs73248905	G	A	-0.097	0.019	1.97E-07	intronic	RALGAPA1	14q13.2	0.07	0.065	0.019	0.148	0.001	0.175
rs137921595	C	T	-0.097	0.019	1.97E-07	intronic	RALGAPA1	14q13.2	0.07	0.065	0.009	0.058	0.001	0.176
rs59698361	A	G	-0.097	0.019	1.97E-07	intronic	RALGAPA1	14q13.2	0.07	0.065	0.009	0.058	0.001	0.176
rs59628537	C	T	-0.097	0.019	1.97E-07	intronic	RALGAPA1	14q13.2	0.07	0.065	0.009	0.058	0.001	0.176
rs12587519	G	A	-0.097	0.019	1.97E-07	intronic	RALGAPA1	14q13.2	0.07	0.065	0.009	0.058	0.002	0.177
rs143998361	G	A	-0.097	0.019	2.09E-07	intronic	RALGAPA1	14q13.2	0.066	0.063	0.007	0.056	0.001	0.177
rs78052351	A	C	-0.1	0.019	2.21E-07	intronic	RALGAPA1	14q13.2	0.07	0.065	0.017	0.132	0.001	0.177
rs57933514	G	A	-0.096	0.019	2.42E-07	intronic	RALGAPA1	14q13.2	0.069	0.065	0.01	0.058	0.001	0.177
rs57490252	A	G	-0.097	0.019	2.48E-07	intronic	RALGAPA1	14q13.2	0.07	0.063	0.016	0.133	0.001	0.176
rs78712778	G	A	-0.096	0.019	2.76E-07	intronic	RALGAPA1	14q13.2	0.068	0.063	0.007	0.059	0.001	0.176
rs75903448	T	G	-0.097	0.019	3.51E-07	intronic	RALGAPA1	14q13.2	0.071	0.067	0.009	0.058	0.001	0.177
rs79683035	A	C	-0.094	0.019	4.07E-07	intronic	RALGAPA1	14q13.2	0.07	0.065	0.009	0.059	0.001	0.177
rs77343697	G	A	-0.094	0.019	7.42E-07	intronic	RALGAPA1	14q13.2	0.053	0.056	0.017	0.15	0.001	0.176
rs76946062	A	T	-0.106	0.021	2.51E-06	intronic	BRMS1L	14q13.2	0.072	0.065	0.007	0.058	0.001	0.18
rs150260495	A	G	-0.087	0.018	2.51E-06	intronic	BRMS1L	14q13.2	0.072	0.065	0.007	0.058	0.001	0.178
rs12587900	C	T	-0.087	0.018	2.51E-06	intronic	BRMS1L	14q13.2	0.072	0.065	0.007	0.017	0.001	0.179
rs76468678	A	T	-0.087	0.018	2.51E-06	intronic	BRMS1L	14q13.2	0.072	0.065	0.007	0.017	0.001	0.179
rs77054567	T	C	-0.087	0.018	3.02E-06	intronic	BRMS1L	14q13.2	0.071	0.065	0.007	0.058	0.001	0.18
rs74635079	A	G	-0.086	0.018	3.02E-06	intronic	BRMS1L	14q13.2	0.071	0.065	0.007	0.058	0.001	0.18
rs59693034	G	A	-0.086	0.018	4.63E-06	intronic	BRMS1L	14q13.2	0.072	0.065	0.007	0.058	0.001	0.18
rs142536166	G	A	-0.084	0.018	4.70E-06	intronic	BRMS1L	14q13.2	0.071	0.065	0.007	0.056	0.001	0.179

rs115188148	C	T	-0.085	0.018	4.82E-06	intronic	BRMS1L	14q13.2	0.071	0.064	0.007	0.058	0.001	0.18
rs79903009	C	A	-0.084	0.018	4.94E-06	intronic	BRMS1L	14q13.2	0.071	0.065	0.007	0.056	0.002	0.179
rs78429311	G	T	-0.084	0.018	4.94E-06	intronic	BRMS1L	14q13.2	0.071	0.065	0.007	0.056	0.001	0.179
rs77902655	G	C	-0.084	0.018	4.94E-06	intronic	BRMS1L	14q13.2	0.071	0.065	0.007	0.058	0.001	0.18
rs77284577	A	G	-0.084	0.018	5.75E-06	intronic	BRMS1L	14q13.2	0.07	0.064	0.007	0.058	0.002	0.18
rs188909493	A	C	-0.085	0.019	6.06E-06	intronic	BRMS1L	14q13.2	0.07	0.064	0.007	0.058	0.001	0.18

REF, reference allele; ALT, alternative allele; Beta, linear regression association coefficient; SE, standard error; TWB, Taiwan Biobank; EAS, East Asian population; AMR, American population; AFR, African population; EUR, European population; SAS, South Asian population.
